# Transient two-dimensional vibrational spectroscopy of an operating molecular machine

**DOI:** 10.1038/s41467-017-02278-6

**Published:** 2017-12-20

**Authors:** Matthijs R. Panman, Chris N. van Dijk, Adriana Huerta-Viga, Hans J. Sanders, Bert H. Bakker, David A. Leigh, Albert M. Brouwer, Wybren Jan Buma, Sander Woutersen

**Affiliations:** 1 Van ’t Hoff Institute for Molecular Sciences, University of Amsterdam, Science Park 904, 1098 XH Amsterdam, The Netherlands; 2 School of Chemistry, University of Manchester, Oxford Road, Manchester, M13 9PL UK; 3 Present Address: Department of Chemistry and Molecular Biology, University of Gothenburg, Box 462, 40530 Gothenburg, Sweden; 4 Present Address: Division of Chemistry and Biological Chemistry, Nanyang Technological University, Singapore, 637371 Singapore

## Abstract

Synthetic molecular machines are promising building blocks for future nanoscopic devices. However, the details of their mechanical behaviour are in many cases still largely unknown. A deeper understanding of mechanics at the molecular level is essential for the design and construction of complex nanodevices. Here, we show that transient two-dimensional infrared (T2DIR) spectroscopy makes it possible to monitor the conformational changes of a translational molecular machine during its operation. Translation of a macrocyclic ring from one station to another on a molecular thread is initiated by a UV pulse. The arrival of the shuttling macrocycle at the final station is visible from a newly appearing cross peak between these two moieties. To eliminate spectral congestion in the T2DIR spectra, we use a subtraction method applicable to many other complex molecular systems. The T2DIR spectra indicate that the macrocycle adopts a boat-like conformation at the final station, which contrasts with the chair-like conformation at the initial station.

## Abstract (web summary)

A deeper understanding of the mechanics of molecular machines is limited by the fast motions which are in the nanosecond or picosecond timescale. Here the authors present a real-time observation of structural changes in a rotaxane-based molecular shuttle by transient two-dimensional infrared spectroscopy.

## Introduction

The development of supramolecular chemistry has made it possible to synthesise externally addressable molecular devices. These synthetic molecular machines are often inspired by the functionality of biological molecular machines^1–4^. In some respects, biological and synthetic molecular machines resemble macroscopic machines. However, many physical concepts governing macroscopic motion are not applicable to microscopic motion^5^. Therefore, the physical principles underlying the operation of nanomachines are actively investigated, both experimentally^6,7^ and theoretically^8^. Synthetic molecular machines are ideal systems to investigate such nanomechanics, due to their simplicity and superior robustness compared to biological molecular machines^9^.

To obtain a deeper understanding of the mechanics of molecular machines, one ideally would like to probe the conformational changes during their operation in real time. Since molecular motions typically occur on the nanosecond or even picosecond time scale, this requires a conformational probe with ultrafast temporal resolution^6^. Transient one-dimensional infrared (henceforth T1DIR) spectroscopy is very suitable for this purpose^10,11^, in particular in the special case that the molecular vibrations are localised on specific chemical bonds and are sensitive to conformation. However, T1DIR spectroscopy only probes changes in the frequencies and absorption intensities of molecular vibrations, and these properties are related to the conformation only in an indirect manner. Transient two-dimensional infrared (T2DIR) spectroscopy is a much more direct probe of structural changes at the molecular level^12–17^. The two-dimensional infrared (2DIR) spectrum of a molecule reveals the couplings between its vibrational modes^18,19^. These couplings are directly related to the relative orientation and distance of the coupled vibrational modes^19^. A 2DIR spectrum therefore provides direct conformational information, similar to 2D-NMR spectroscopy, but with a time resolution of typically 1 ps or less (determined by the vibrational free-induction decay and the IR pulse duration). In time-resolved 2DIR spectroscopy, one triggers a conformational change (e.g. by a UV-induced photoisomerisation or a temperature jump), and subsequently records 2DIR spectra at specific time delays with respect to the trigger. By recording a series of 2DIR spectra at increasing time delays, a molecular movie of the conformational changes can be obtained^20^. T2DIR spectroscopy is a relatively new method, but has already been applied successfully to investigate photo-^12,13^ and temperature-induced^15,21^ folding of peptides, tautomerisation of organic compounds^16^ and structural rearrangements in catalytic complexes^14^. Here, we use T2DIR spectroscopy to investigate the structural changes in a molecular machine during its operation cycle.

The molecular machine investigated here is a rotaxane-based molecular shuttle. The shuttling macrocycle and its two docking stations each contain amide groups, and the corresponding vibrational modes provide excellent localised vibrational chromophores^11,22^, which are known to couple strongly and in a distance- and orientation-dependent manner^23,24^. Thus, the relative distance and orientation of the components of the molecular shuttle can be probed by measuring the interactions between their respective CO-stretch and amide I vibrations;^25^ and by triggering the shuttling motion and recording a series of 2DIR snapshots, we can track the conformational changes as the shuttle progresses through its operation cycle.

We find that the cross peaks in the T2DIR spectra of the molecular shuttle recorded at different stages of its operation cycle directly reveal the proximity of the shuttling macrocycle to the different stations on the thread. Interpreting the T2DIR spectra using quantum-chemical calculations, we find evidence that the macrocycle adopts two distinct conformations depending on the station to which it is hydrogen bonded. To isolate the macrocycle/thread cross peaks from the other contributions to the T2DIR spectrum, we use a method for decomposing the congested equilibrium- and transient-2DIR spectra that exploits the presence of a localised electronic chromophore in the investigated molecule. We believe this method may be useful in other cases as well, especially because spectral congestion will occur more frequently as larger and more complex systems are investigated with 2DIR spectroscopy.

## Results

### Photocycle of the molecular shuttle

**Fig. 1:**
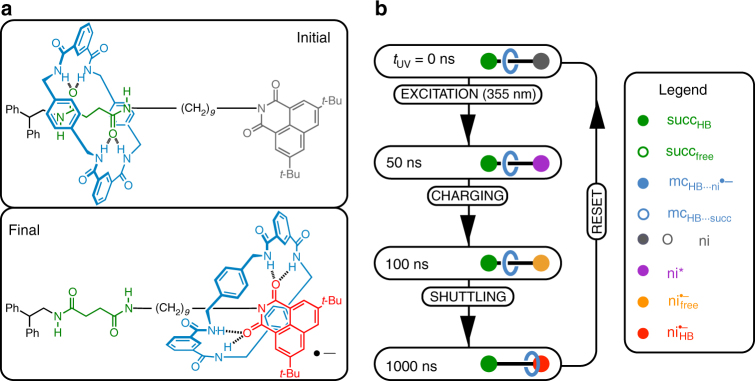
Structure and operation cycle of the molecular shuttle. **a** Chemical structure of the molecular shuttle. **b** Photochemical operation cycle. The operation of the shuttle is initiated by a UV light pulse (EXCITATION), which causes a reduction of the ni^*^ by an external electron donor (CHARGING). The subsequent translational motion of the macrocycle occurs between 100 and 1000 ns (SHUTTLING). The shuttle is RESET after charge recombination. The legend colours correspond to the coloured components of the molecular shuttle

The chemical structure of the rotaxane-based shuttle is shown in Fig. 1a. The thread contains two stations that each can form hydrogen bonds with the macrocycle. The light-induced operation of the molecular shuttle is a three-stage process (Fig. 1b)^11^. Prior to the absorption of UV light, the macrocyclic ring (mc, blue in Fig. 1) is predominantly hydrogen bonded to the succinamide docking station (succ, green in Fig. 1). Absorption of a 355 nm photon leads to electronic excitation of the naphthalimide station (ni, grey in Fig. 1), followed by intersystem crossing to the triplet state (ni^*^, purple in Fig. 1). Subsequently, an electron donor (1, 4-diazabicyclo[2.2.2]octane) that is also present in solution reduces the ni^*^ state to a radical anion (ni^•−^, red in Fig. 1). The macrocycle can form hydrogen bonds to the ni^•−^ station that are much stronger than those to the succ station^26^, and this inversion of the comparative hydrogen-bond strengths drives the translation of the macrocycle across the thread. After charge recombination (which occurs on a ~100-μs time scale), the macrocycle shuttles back to the succ station^11^.

### Steady-state and time-resolved 1D and 2D spectra

**Fig. 2:**
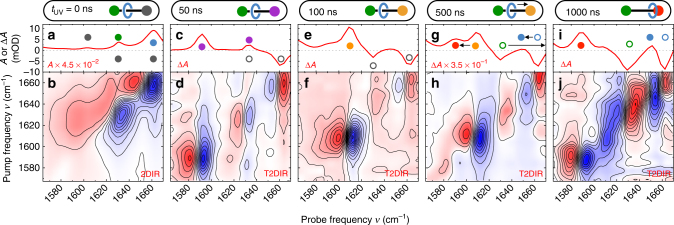
Transient 2DIR spectra during the operation cycle of the molecular machine. **a**, **c**, **e**, **g**, **i** are the absorption (*A*) and change in absorption (Δ*A*) spectra; **b**, **d**, **f**, **h**, **j** are the corresponding 2DIR (Δ*A*) and T2DIR (ΔΔ*A*) spectra measured with parallel (||) polarisations of the IR-pump and IR-probe pulses. The coloured closed and open circles correspond to the labelled components of the molecular shuttle depicted schematically above the graphs. Arrows indicate spectral shifts caused by the shuttling of the macrocycle

In the following, we will present four types of spectra: (1) steady-state infrared (1DIR) absorption spectra, which provide information on the molecular shuttle in thermal equilibrium. (2) UV-pump 1DIR-probe difference-absorption spectra (T1DIR), that show the UV-induced time-dependent changes in the vibrational absorptions (Δ*A*). T1DIR spectra comprise positive and negative contributions which arise from the vibrational modes of the UV-induced transient species and depleted electronic ground state, respectively. (3) Steady-state 2DIR spectra, which reveal the vibrational couplings in the electronic ground state. (4) T2DIR spectra, that show the UV-induced time-dependent changes in the 2DIR spectrum. The T2DIR spectra comprise contributions from the UV-induced transient species and contributions due to the electronic ground-state depletion. Both in the T1DIR and T2DIR spectra, the signals associated with the depleted electronic ground-state species have the opposite sign of those of the UV-generated transient species. Specifically, the T2DIR spectrum is obtained by subtraction of the steady-state 2DIR signal Δ*A*_2DIR_ from the UV-pumped signal Δ*A*_UV,2DIR_:

1 $${\mathrm{\Delta \Delta }}A\left( {\omega _{{\mathrm{probe}}},\omega _{{\mathrm{pump}}},t_{{\mathrm{UV}}}} \right) = {\mathrm{\Delta }}A_{{\mathrm{UV,2DIR}}}\left( {\omega _{{\mathrm{probe}}},\omega _{{\mathrm{pump}}},t_{{\mathrm{UV}}}} \right) \\ - {\mathrm{\Delta }}A_{{\mathrm{2DIR}}}\left( {\omega _{{\mathrm{probe}}},\omega _{{\mathrm{pump}}}} \right),$$

where *t*_UV_ is the waiting time between the triggering UV pulse and the probing 2DIR pulse pair. Coherent interactions caused by temporal overlap of the IR pulse-pair are avoided by using a fixed 1-ps delay between the IR-pump and IR-probe pulses. Figure 2b shows the four categories of IR spectra for the rotaxane, observed at different UV–IR waiting times. Transient and linear absorptions are labelled with solid circles which match the colours of the rotaxane components in Fig. 2a. Open circles are used for bleached ground-state contributions. The assignment of the peaks is based on previous 1D-spectroscopy experiments^11^. Coordinates (*ν*_pump_, *ν*_probe_) of spectral features in the 2DIR and T2DIR spectra refer to the negative feature for both diagonal and cross peaks.

### Molecular shuttle in thermal equilibrium

In thermal equilibrium, the macrocyclic ring is hydrogen bonded to the succ station (Fig. 1a). The imide CO-stretch and amide I absorptions of the three components of the shuttle (macrocycle, initial and final stations) are observed in the 1DIR spectrum (Fig. 2a, *t*_UV_ = 0 ns). Supplementary Table 1 lists the assignments of the IR absorptions. The amide I vibrational modes associated with the macrocycle hydrogen bonded to the succ station (mc_HB⋯succ_, 1663 cm^−1^) and the hydrogen-bonded succ station (succ_HB_, 1632 cm^−1^) are the most relevant probes for determining the conformation of the macrocycle when bound to the initial station.

Figure 2b shows the thermal-equilibrium (electronic ground state) 2DIR spectrum of the molecular shuttle. This spectrum shows the difference Δ*A* in IR absorption induced by a spectrally narrow (16 cm^−1^ FWHM) tunable IR pump pulse, as a function of the probe frequency and of the centre frequency of the pump pulse (double-resonance 2DIR spectroscopy). When the pump frequency is resonant with one of the vibrational modes, the *v* = 1 state of this mode is populated, resulting in a signal on the diagonal peak of the 2DIR spectrum. Each diagonal peak comprises a negative (blue) and positive (red) Δ*A* part: the former is caused by vibrational ground-state depopulation and *v* = 1 → 0 stimulated emission, the latter by the *v* = 1 → 2 transition (which occurs at a lower frequency due to anharmonicity). Two diagonal features at (*ν*_pump_, *ν*_probe_) = (1663 cm^−1^, 1663 cm^−1^) and (1632 cm^−1^, 1632 cm^−1^) are observed in the 2DIR spectrum, which correspond to the mc_HB⋯succ_ and succ_HB_ resonances, respectively. Off-diagonal peaks resembling the diagonal features appear in the spectrum at (*ν*_pump_, *ν*_probe_) = (*ν*_*i*_, *ν*_*j*_) and (*ν*_*j*_, *ν*_*i*_) when two modes *i* and *j* are coupled. The cross peak observed between the mc_HB⋯succ_ and succ_HB_ bands at (1663 cm^−1^, 1632 cm^−1^) is an indication of the coupling (and thus proximity) of the succ station and macrocycle CO groups. However, the mc_HB⋯succ_ and succ_HB_ modes have significant spectral overlap with one of the naphthalimide aromatic-stretch (ni_Ar_) modes and with the antisymmetric naphthalimide CO-stretch (ni_as_) mode. The latter two ni modes are strongly coupled (because they are located in the same molecular moiety), giving give rise to a strong ni_Ar_/ni_as_ cross peak. Due to the spectral overlap, we cannot obtain the separate contributions of the ni_Ar_/ni_as_ and succ_HB_/mc_HB⋯succ_ cross peaks from the congested 2DIR spectrum. Below, we will demonstrate how T2DIR can be used to solve this 2D-spectral congestion problem.

### Activating the shuttle

Within a few nanoseconds after the absorption of a UV photon, the naphthalimide group (ni^*^) is in its triplet state due to rapid intersystem crossing. Figure 2c shows the T1DIR spectrum after completion of this first step (*t*_UV_ = 50 ns) of the operating cycle of the molecular shuttle. In this transient spectrum, both ni^*^ absorption and ni ground-state bleaching peaks are observed. Comparing the *t*_UV_ = 50 ns T2DIR spectra of the rotaxane (Fig. 2d) and the bare thread, we find these spectra to be identical (refer to Supplementary Fig. 1 for the structure and spectral comparison, and see Supplementary Note 1 for a discussion). This spectral similarity indicates that at *t*_UV_ = 50 ns, no changes have yet occurred in the hydrogen-bonded mc:succ conformation at the initial station.

With increasing waiting time (see *t*_UV_ = 100 ns in Fig. 2e), we observe a spectral change associated with the ni^*^ station accepting an electron from the external donor (ni^*^ → ni^•−^): the disappearance of the antisymmetric and symmetric CO-stretch modes (respectively ${\mathrm{ns}}_{{\mathrm{as}}}^*$ and ${\mathrm{ni}}_{\mathrm{s}}^*$) of the ni^*^, and the appearance of the (non-hydrogen-bonded) symmetric CO-stretch vibration $\left( {{\mathrm{ni}}_{{\mathrm{free}}}^{ \bullet - }} \right)$ of the ni^•−^ species at 1613 cm^−1^ (the radical-anion asymmetric CO-stretch vibration absorbs at a frequency outside the investigated spectral range^11^). Figure 2f shows the T2DIR spectrum at *t*_UV_ = 100 ns. The most notable diagonal feature is due to the ${\mathrm{ni}}_{{\mathrm{free}}}^{ \bullet - }$ mode, which at this waiting time does not interact with the other modes in the investigated spectral window. This lack of vibrational couplings indicates that at this waiting time, the ni^•−^ station is still spatially separated from the other functional units (succ, mc) of the molecular shuttle. The fact that all signals in the T2DIR spectrum arise from the ni unit indicates that at this waiting time the macrocyclic ring and the succ station still have the same conformation as before the UV trigger (so that ΔΔ*A* = 0 for these components).

### Unravelling spectral congestion

**Fig. 3:**
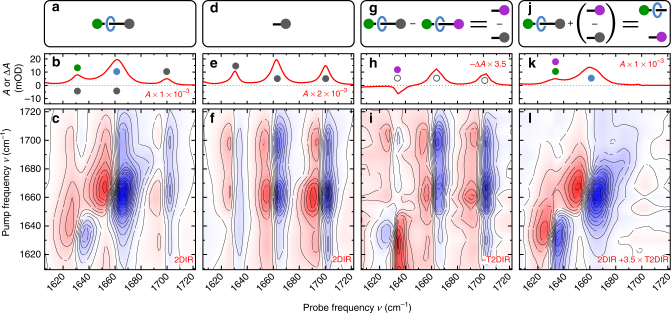
Unravelling a congested 2DIR spectrum with T2DIR. **a**, **d**, **g**, **j** are schematic representations of the components of the molecular shuttle belonging to the spectra. In the 1DIR (**b**) and 2DIR (**c**) spectra of the molecular shuttle, the peaks due to vibrations of the ni station have significant overlap with the peaks due to the vibrations of the succ station and the macrocycle. The 1DIR (**e**) and 2DIR (**f**) spectra of the isolated ni station, which only contain absorption peaks due to vibrations of the ni station. At 50 ns after UV excitation, the absorption change observed in the T1DIR (**h**) and T2DIR (**i**) spectra are due only to changes in the vibrational frequencies in the ni station. The T2DIR spectrum in (**i**) has been multiplied by −1 for better comparison with the 2DIR spectrum of the chemically isolated ni stopper in (**f**). The constructed ni-free 1DIR (**k**) and 2DIR (**l**) spectra of the molecular shuttle show the absorption peaks due to the succ station and macrocycle. The coloured closed and open circles correspond to the labelled components of the molecular shuttle in Fig. 1b. The 2D spectra are recorded with perpendicular (⊥) polarisations of the IR-pump and IR-probe pulses

At *t*_UV_ delays prior to shuttling, the UV excitation only affects the ni component of the rotaxane. We can use this property to isolate the overlapping contributions of the features associated with the macrocycle, and ni and succ stations in the 2DIR spectrum (Fig. 3c). The vibrations related to the ni station are selectively depleted (see Supplementary Fig. 2 and Supplementary Note 2), whereas the vibrations of the remainder of the shuttle (the modes associated with the macrocycle and the succ station) cancel according to Eq. 1, and therefore do not appear in the T2DIR spectrum. The T2DIR signal therefore only comprises the UV-induced 2DIR signal which belongs to the ni^•−^ component and an inverted 2DIR signal due to the depleted electronic ground state of the ni component. This selectivity due to the UV excitation is confirmed by the similarity of the steady-state 2DIR spectrum of a solution containing only the ni^•−^ station in its electronic ground state (Fig. 3f) and the depletion-T2DIR spectrum of the complete shuttle multiplied by −1 (Fig. 3i).

Highlighting specific contributions in a congested 2DIR spectrum through selective depletion is a generally applicable method: the 2DIR spectrum of an excited moiety of a molecular system can be measured separately from that of the other parts of the system provided that the external stimulus (be it UV- visible excitation, or otherwise) generates a sufficient change in the 2DIR signal of the moiety. The depletion will manifest as an inverted 2DIR signal in the T2DIR response. The method is also applicable if the UV/Vis excitation leads to non-reversible changes, such as photobleaching of particular moieties of interest: the signals belonging to the remainder of the molecule are unchanged and therefore cancel in the T2DIR spectrum, and one only observes the depleted contributions of the photobleached moiety. Practical aspects to be considered for the depletion method to work are (1) the signal-to-noise ratio and (2) the polarisation dependence of the signals. (1) If the 2DIR spectrum of depleted part of the molecule has a much larger amplitude than that of the remainder of the molecule, the difference-2DIR spectrum will be difficult to measure. Even if the 2DIR spectra have similar amplitudes, one has to average longer to obtain the same signal-to-noise ratio in the difference measurement as in the raw 2DIR spectra^27^. On the positive side, since the difference is taken between two spectra that differ only in *t*_UV_ (a parameter that is modulated on a time scale of ~1 Hz in our experiments), systematic errors due to, e.g., beam drift can to some extent be cancelled in the depletion-T2DIR spectrum. (2) Excitation by the linearly polarised UV pulse generates an anisotropic distribution of excited molecules. For cancellation of the depleted 2DIR spectrum to occur, the waiting time *t*_UV_ should be sufficient for the UV-excited molecules to scramble this anisotropy by rotational diffusion, so that the cancelling contributions in Eq. (1) both arise from isotropic distributions of molecules, and thus have the same IR-pump/IR-probe polarisation dependence^28^. This condition is met in our experiments, since the orientational relaxation time of the rotaxane is <0.5 ns (see Supplementary Note 3), much shorter than our *t*_UV_ delays. If the orientational scrambling was slower than *t*_UV_, the equivalent of an isotropic depletion-T2DIR spectrum can still be obtained for parallel IR–IR polarisations by setting the UV and IR polarisations at the magic-angle (54.7°)^28^, but this is not possible for the perpendicular IR–IR polarisations. In the next section, we will demonstrate how we can separate the 2DIR contribution of the residual (non-excited) parts of the molecular device from the full 2DIR spectrum.

### The equilibrium conformation of the shuttle

We can use the selectivity of the UV excitation in the T2DIR spectrum to cancel out the ni signals in the steady-state 2DIR spectrum, thereby isolating the signals associated with initial docking station and the macrocyclic ring. To achieve this, we only need to subtract the properly scaled T2DIR-depletion signal from the total steady-state 2DIR spectrum. To illustrate the principle, we first obtain the 1DIR spectrum of the macrocycle and succ station by eliminating the contribution of the ni station: the 1DIR signal of the rotaxane and that of the free ni station (Fig. 3b, e, respectively) are normalised on the amplitude of the ni_s_ absorption. Subsequently, the spectra are subtracted from one another to generate the 1DIR spectrum (Fig. 3k) containing purely the vibrations of the succinamide station succ_HB_ (amide I mode of the hydrogen-bonded succ station) and macrocycle mc_HB⋯succ_ (amide I mode of the macrocycle hydrogen bonded to the succ station). We can use an analogous method to obtain the ni-free 2DIR signal. We add the appropriately scaled *t*_UV_ = 50 ns T2DIR spectrum (Fig. 3i) to the 2DIR spectrum of the ground state (Fig. 3c), eliminating the absorptions associated with the ni station (see Supplementary Note 4 for the details of the scaling procedure; see also ref. ^29^, where we used a separately measured steady-state 2DIR spectrum of the ni station to eliminate the ni contribution to the 2DIR spectrum of the shuttle). Two diagonal features at (1632 cm^−1^, 1632 cm^−1^) and (1663 cm^−1^, 1663 cm^−1^) are observed in the resulting 2DIR spectrum (Fig. 3l), which belong to the overlapping succ_HB_ amide I and ${\mathrm{ni}}_{\mathrm{s}}^*$ imide-stretch modes in addition to the mc_HB⋯succ_, respectively. The presence of the ${\mathrm{ni}}_{\mathrm{s}}^*$ signal is a consequence of the addition of the T2DIR spectrum. However, since the triplet species do not absorb above 1632 cm^−1^ in this spectral range (Fig. 3h), the off-diagonal signals at (1632 cm^−1^, 1663 cm^−1^) and (1663 cm^−1^, 1632 cm^−1^) (Fig. 3i) arise solely from the interaction (coupling) of the succ station with the macrocyclic ring.

**Fig. 4:**
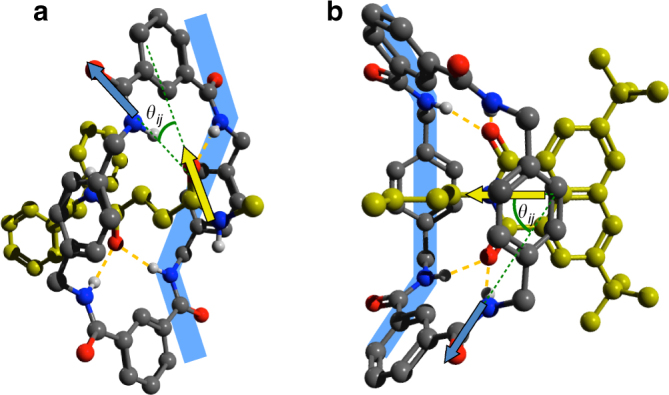
Conformations of the macrocyclic ring, hydrogen bonded to the initial and final station of the molecular shuttle. **a** Optimised geometry of the macrocyclic ring, hydrogen bonded to the succ station. **b** Optimised geometry of the macrocyclic ring, hydrogen bonded to the ni^•−^ station. In both cases, the conformations are optimised at the B3LYP/6-31G(d) level. The C atoms of the stations are coloured yellow, and the macrocycle⋯station hydrogen bonds are indicated by dashed orange lines. The transition-dipole-moment vectors, whose relative angle *θ*_*ij*_ can be determined from the cross-peak anisotropies in the (T)2DIR spectra, are indicated by green arrows, their protractions by green dashed lines. The macrocycle conformation is indicated in light blue

We can determine the geometry of the molecular device in thermal equilibrium from the mc/succ cross-peak anisotropy (see Supplementary Fig. 3) in the ni-free 2DIR signal. The experimentally observed anisotropy *R*_*ij*_ of a cross peak between oscillators *i* and *j* is directly related to the angle *θ*_*ij*_ between their transition-dipole moments:^19^

2 $$R_{ij} = \frac{{{\mathrm{\Delta }}A_{||} - {\mathrm{\Delta }}A_ \bot }}{{{\mathrm{\Delta }}A_{||} + 2{\mathrm{\Delta }}A_ \bot }} = \frac{{3\,{\mathrm{cos}}^2\theta _{ij} - 1}}{5},$$

where Δ*A*_⊥_ and Δ*A*_||_ are the cross-peak intensities for perpendicular and parallel polarisations of the IR pulse pair, respectively. We use Eq. (2) to obtain *θ*_succ/mc_ = 55±3° and *θ*_mc/succ_ = 42 ± 3° from the 2DIR signal at *ν*_pump_ = *ν*_succ_ and *ν*_pump_ = *ν*_mc_, respectively. The uncertainties in these values are based on the error bars of the baseline-corrected data (based on a Singular Value Decomposition method^30^, see Supplementary Methods and Supplementary Fig. 4 for details of the baseline-correction procedure), and were estimated using error propagation^27^. These statistical estimates of the uncertainties should be regarded as lower limits of the real uncertainties, as they do not take into account systematic experimental errors and certain assumptions in the exciton model used to analyse the spectra^19^. To see if the angle between the succ_HB_ and mc_HB⋯succ_ transition-dipole moments as obtained from the 2DIR experiment is realistic, we use a density-functional theory (DFT)-optimised structure (at the B3LYP/6-31G(d) level, see Supplementary Fig. 5, Supplementary Table 2 and Supplementary Discussion for the calculated frequencies and details of the calculation) of a model compound mimicking the initial state (Fig. 4a) to extract the angle between the succ_HB_ and mc_HB⋯succ_ transition-dipole moments. These DFT calculations reproduce the transient vibrational spectra very well (see Supplementary Discussion and Supplementary Fig. 5). From the DFT-optimised structure, we obtain an angle of 38° between the succ_HB_ and mc_HB⋯succ_ transition-dipole moments which agrees reasonably well with the experimental value (see Supplementary Note 5 and Supplementary Fig. 6 for details of the relationship between *θ*_*ij*_ and the CO group geometry). This angle corresponds to the chair-like conformation (Fig. 4a) that we observed previously in a short (non-shuttling) succinamide-based rotaxane using 1- and 2-colour 2DIR spectroscopy^25,31^.

### Shuttling motion

In the T1DIR spectra at *t*_UV_ = 500 and 1000 ns (Fig. 2j), we observe the disappearance of the ${\mathrm{ni}}_{{\mathrm{free}}}^{ \bullet - }$ absorption and the appearance of a red-shifted absorption at 1592 cm^−1^. This red shift is due to the formation of the strong hydrogen bonds between the macrocycle and the ni^•−^ station^11,22^. The concomitant breaking of the hydrogen bonds between the macrocycle and the succ station causes a negative absorption change at 1632 cm^−1^, the amide I frequency of the hydrogen-bonded succ station (the corresponding increase of the non-hydrogen-bonded succ amide I mode is outside our investigated spectral range). The stronger hydrogen bonding to the ni^•−^ station as compared to the succ station causes the mc amide I mode frequency to red shift upon shuttling, from 1663 to 1654 cm^−1^.

### Eliminating ground-state signals from the T2DIR spectrum

**Fig. 5:**
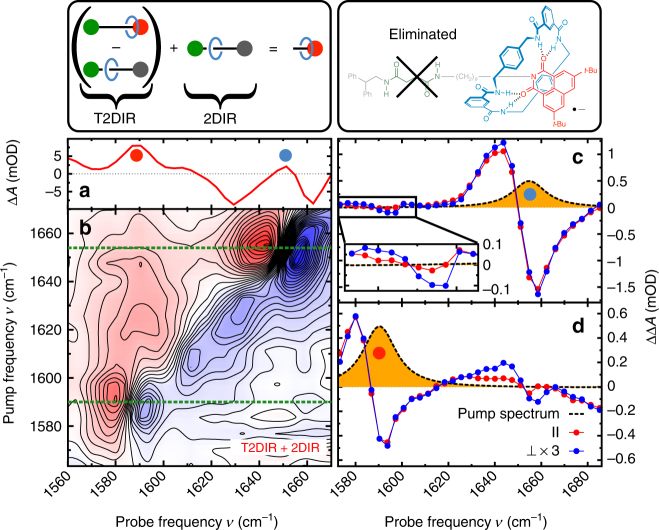
Isolating the switched state of the molecular shuttle. **a** T1DIR spectrum of the molecular shuttle at *t*_UV_ = 1000 ns. The colours of the dots indicating the different peaks correspond to the colours of the components of the molecular shuttle in the panels above, and in Fig. 2a. **b** Depletion-corrected T2DIR spectrum (ΔΔ*A*) of the molecular shuttle at *t*_UV_ = 1000 ns. **c** Slice of the depletion-corrected T2DIR spectrum (ΔΔ*A*). The yellow peak is the power spectrum of the pump pulse, centred at 1654 cm^−1^. **d** Slice of the depletion-corrected T2DIR spectrum (ΔΔ*A*). The yellow peak is the power spectrum of the pump pulse, centred at 1592 cm^−1^. In each spectrum, the red curve is the spectrum measured with parallel (||) polarisations of the IR-pump ad IR-probe pulses. The blue curve is the spectrum measured with perpendicular (⊥) polarisations of the IR-pump and IR-probe pulses. The perpendicular signal in both T2DIR slices is multiplied by 3 for better comparison with the parallel signal

The T2DIR spectrum at *t*_UV_ = 1000 ns is significantly more complex than at the earlier *t*_UV_ delays. The shuttling causes the amide I and CO-stretch frequencies of all three components of the shuttle (macrocycle, and succ and ni stations) to change. As a consequence, the ground-state depletion signal at *t*_UV_ = 1000 ns (Fig. 2f) is a reflection of the 2DIR spectrum of the entire rotaxane as opposed to the ni station-specific ground-state depletion signal measured at *t*_UV_ ≤ 100 ns. The addition of the properly scaled ground-state 2DIR response to the T2DIR signals results in the cancellation of signals belonging to the depleted ground state, and in this way we can isolate the T2DIR spectrum of the switched conformation of the molecular device from the total T2DIR spectrum (i.e. the second term in Eq. (1) is eliminated; see Supplementary Note 6 and Supplementary Fig. 7 for details of the scaling procedure). Figure 5b shows the depletion-free T2DIR spectrum: the ground-state bleaching is eliminated, and the 2DIR spectrum is effectively that of a sample containing only photoexcited molecular shuttles in which the macrocycle has arrived at the final, ni^•−^ station. In the simplified spectrum, we observe two diagonal features: (1592 cm^−1^, 1592 cm^−1^) which is due to the ${\mathrm{ni}}_{{\mathrm{HB}}}^{ \bullet - }$ mode (imide CO-stretch vibration of the hydrogen-bonded ni^•−^ station), and (1654 cm^−1^, 1654 cm^−1^) which is due to the ${\mathrm{mc}}_{{\mathrm{HB}} \cdots {\mathrm{ni}}^{ \bullet - }}$ mode (amide I vibration of the macrocycle hydrogen bonded to the ni station). At *t*_UV_ = 1000 ns, a small fraction of the macrocycles in the sample still reside at the succ station, observable as a low-intensity Δ*A* at 1613 cm^−1^ (${\mathrm{ni}}_{{\mathrm{free}}}^{ \bullet - }$ vibration). Additionally, a small positive off-diagonal feature at ($\nu _{{\mathrm{ni}}_{{\mathrm{HB}}}^{ \bullet - }}$, $\nu _{{\mathrm{mc}}_{{\mathrm{HB}} \cdots {\mathrm{ni}}^{ \bullet - }}}$) = (1654 cm^−1^, 1580 cm^−1^) indicates the proximity of the ni^•−^ station and the macrocycle. This cross-peak feature is a direct demonstration that the molecular device has successfully switched. We expect to observe a complementary cross peak at (*ν*_pump_, *ν*_probe_) = ($\nu _{{\mathrm{ni}}_{{\mathrm{HB}}}^{ \bullet - }}$, $\nu _{{\mathrm{mc}}_{{\mathrm{HB}} \cdots {\mathrm{ni}}^{ \bullet - }}}$), but this is difficult to observe with our signal-to-noise ratio. We therefore conducted a separate experiment in which we record two relevant horizontal slices of the T2DIR spectrum with much greater precision. The corrected (depletion-free) T2DIR slices at *t*_UV_ = 1000 ns for parallel and perpendicular polarisations of the IR-pulse pair are shown in Fig. 5c, d (the uncorrected T2DIR spectra are shown in the Supplementary Fig. 8). The ΔΔ*A* signal observed at *ν*_pump_ = $\nu _{{\mathrm{mc}}_{{\mathrm{HB}} \cdots {\mathrm{ni}}^{ \bullet - }}}$ (Fig. 5c) is the most prominent indication of the coupling between the ni^•−^ station and macrocycle. A resonant feature is observed at *ν*_probe_ = 1659 cm^−1^ and 1644 cm^−1^ (${\mathrm{mc}}_{{\mathrm{HB}} \cdots {\mathrm{ni}}^{ \bullet - }}$ mode). Additionally, a polarisation-dependent ΔΔ*A* cross peak is observed at *ν*_probe_ = 1592 and 1580 cm^−1^ which arises from the coupled ${\mathrm{ni}}_{{\mathrm{HB}}}^{ \bullet - }$ and ${\mathrm{mc}}_{{\mathrm{HB}} \cdots {\mathrm{ni}}^{ \bullet - }}$ modes (see inset for a magnification of the signal). Figure 5d displays the *ν*_pump_ = $\nu _{{\mathrm{ni}}_{{\mathrm{HB}}}^{ \bullet - }}$ T2DIR signal. Besides a ${\mathrm{ni}}_{{\mathrm{HB}}}^{ \bullet - }$ diagonal peak at *ν*_probe_ = 1592 and 1580 cm^−1^, a polarisation-dependent ΔΔ*A* signal at *ν*_probe_ = 1654 and 1644 cm^−1^ corresponding to the ${\mathrm{mc}}_{{\mathrm{HB}} \cdots {\mathrm{ni}}^{ \bullet - }}$ mode is observed, in addition to the ${\mathrm{ni}}_{{\mathrm{HB}}}^{ \bullet - }$ diagonal peak at *ν*_probe_ = 1592 and 1580 cm^−1^. The off-diagonal feature is the complementary cross peak to that in Fig. 5c, an additional confirmation that the ${\mathrm{mc}}_{{\mathrm{HB}} \cdots {\mathrm{ni}}^{ \bullet - }}$ and ${\mathrm{ni}}_{{\mathrm{HB}}}^{ \bullet - }$ modes are coupled.

The cross peaks between the macrocycle and the final station on the thread are a direct demonstration that shuttling of the macrocycle has occurred. These cross peaks are somewhat similar to the NOESY cross peaks used to demonstrate spatial proximity of specific nuclear spins of proteins in 2D-NMR spectroscopy. Due to the much better time resolution of T2DIR compared to 2D-NMR, the time dependence of the cross-peak intensity can be used to track the arrival of the shuttle in real time. This can be seen at an intermediate waiting time (*t*_UV_ = 570 ns, Fig. 2g, h), where the ${\mathrm{ni}}_{{\mathrm{HB}}}^{ \bullet - }$ and ${\mathrm{ni}}_{{\mathrm{free}}}^{ \bullet - }$ absorptions are at half their maximum intensity, indicating that the fractions of macrocycles hydrogen bonded to the ni^•−^ and to the succ stations are approximately equal. The T2DIR response at *t*_UV_ = 570 ns is therefore an average of the T2DIR signals at *t*_UV_ = 1000 and 100 ns. The intermediate T2DIR spectrum illustrates that by measuring T2DIR spectra for a series of *t*_UV_ values, we can observe the hydrogen-bond formation between the macrocycle and the ni^•−^ station in a time-resolved manner.

### The conformation in the switched state

To determine the angle between the transition-dipole moments of the coupled ${\mathrm{ni}}_{{\mathrm{HB}}}^{ \bullet - }$ and ${\mathrm{mc}}_{{\mathrm{HB}} \cdots {\mathrm{ni}}^{ \bullet - }}$ modes, we can use the anisotropy of the cross peak arising from the coupling between these two vibrations. The vibrational coupling gives rise to a pair of cross peaks at $\left( {\nu _{{\mathrm{ni}}_{{\mathrm{HB}}}^{ \bullet - }},\nu _{{\mathrm{mc}}_{{\mathrm{HB}} \cdots {\mathrm{ni}}^{ \bullet - }}}} \right)$ and $\left( {\nu _{{\mathrm{mc}}_{{\mathrm{HB}} \cdots {\mathrm{ni}}^{ \bullet - }}},\nu _{{\mathrm{ni}}_{{\mathrm{HB}}}^{ \bullet - }}} \right)$, that can be observed in the slices at *ν*_pump_ = 1654 and 1592 cm^−1^, respectively. The anisotropy of each of the two complementary cross peaks can be used to determine *θ*_*ij*_. To determine the cross-peak anisotropy, we use the ΔΔ*A*_||_ and ΔΔ*A*_⊥_ signals at different probe frequencies in the cross-peak range. For *ν*_pump_ = 1654 cm^−1^, we use *ν*_probe_ = 1581, 1585, 1588 and 1598 cm^−1^; and for *ν*_pump_ = 1592 cm^−1^, we use *ν*_probe_ = 1648, 1652 and 1659 cm^−1^. The average angles obtained in this way are *θ*_*ij*_ = 52 ± 9° when *ν*_pump_ = $\nu _{{\mathrm{mc}}_{{\mathrm{HB}} \cdots {\mathrm{ni}}^{ \bullet - }}}$ and *θ*_*ij*_ = 48 ± 4° for *ν*_pump_ = $\nu _{{\mathrm{ni}}_{{\mathrm{HB}}}^{ \bullet - }}$. The uncertainties in these values were obtained from the error bars in the baseline-corrected data using error propagation^27^ (see Supplementary Methods and Supplementary Fig. 9 for details of the baseline-correction procedure). As for the angle reported above for the equilibrium conformation, the uncertainties should be regarded as lower limits of the actual uncertainties since the data analysis does not take systematic-error contributions into account.

To complement the T2DIR experiments, we perform DFT calculations on a model compound comprising the macrocyclic ring, a short thread and the ni^•−^ unit. In the optimised structure (Fig. 4b), the macrocycle adopts a boat-like conformation to optimise the hydrogen bonding to the ni^•−^ station (resulting in four NH⋯CO inter-component hydrogen bonds). In the calculated structure, the ${\mathrm{ni}}_{{\mathrm{HB}}}^{ \bullet - }$ and ${\mathrm{mc}}_{{\mathrm{HB}} \cdots {\mathrm{ni}}^{ \bullet - }}$ transition-dipole moments are at an angle of 46.7° (for details of the relationship between *θ*_*ij*_ and the CO group geometry, see Supplementary Note 5 and Supplementary Fig. 6), in good agreement with the value that we estimated from the T2DIR cross-peak anisotropies.

## Discussion

In the above, we have shown that the conformational changes of a molecular device can be followed during its operation with the combined temporal and structural resolution of T2DIR. T2DIR spectroscopy can be used to investigate other molecular machines in a similar manner, with certain practical boundary conditions: (1) the motion must be externally triggered and (2) the number of IR-active modes in the investigated spectral region should be sufficiently small to enable unambiguous interpretation of the spectra. This somewhat limits the applicability for peptide-based molecular devices, although isotope-substitution can be used to limit the spectral congestion. (3) Since the cross-peak intensities decay rapidly with increasing distance (as 1/*R* ^6^ for through-space dipolar coupling), the IR chromophores in the moving parts must be in close contact to give rise to observable cross peaks.

From the cross peaks in the T2DIR spectrum, we can directly observe the proximity of the macrocycle to the initial or final stations on the thread, and even obtain quantitative information about the macrocycle conformation in the initial and (short-lived) final state of the molecular shuttle. In addition, we demonstrate that we can obtain the electronic ground-state 2DIR spectrum of a UV/Vis chromophore from its optically excited ground-state depletion spectrum, which can be a useful method to simplify complex 2DIR spectra. In fact, using this subtraction method was essential to determine the macrocycle conformation in the equilibrium state, since the conventional 2DIR spectrum would have been too congested to do so. A similar procedure can be used in any molecular system that contains a moiety whose electronic state can be modified (resulting in a large shift of the vibrational frequencies), for instance by photo- or electrochemical methods.

## Methods

### Sample synthesis and preparation

We synthesised the rotaxane as described previously^32^. The spectroscopic experiments are performed on a solution of rotaxane (5 × 10^−4^ M) and 1, 4-diazabicyclo[2.2.2]octane (DABCO, 5 × 10^−2^ M) in CD_3_CN (Eurisotop, >99.8% D purity). The solvent and DABCO have no absorption bands in the investigated IR-spectral region. To remove dissolved oxygen, argon is bubbled through the solution for >15 min prior to the experiments. The sample is kept at 70 °C in a thermostated IR cell which consists of two CaF_2_ windows separated by a 5-mm spacer. To avoid photochemical degradation and heating due to accumulated UV-pump pulses, we move the sample cell in a Lissajous figure using two computer-controlled translation stages.

### T2DIR and steady-state measurements

The T2DIR spectrometer is described in detail in the Supplementary Methods, see Supplementary Fig. 10 for the electronic timing. In brief, a UV-pump pulse (355 nm, 3.6 ns) is used to induce the photochemical reduction of the molecular shuttle at a 100 Hz repetition rate. The T1DIR and T2DIR spectra are obtained with a 50 Hz repetition rate at different times after UV excitation. T2DIR spectra were measured by scanning a narrow band pump pulse (16 cm^−1^, 800 fs FWHM) using a Fabry-Perot interferometer^25^. The absorption changes were monitored using frequency-dispersed detection of the broad-band probe pulses using a 2 × 32 pixel HgCdTe array detector. The 2DIR spectra were obtained with a repetition rate of 450 Hz in a similar fashion without the UV pump. Conventional (steady-state) Fourier-transform infrared (1DIR) spectra are measured with a Bruker Vertex 70 spectrometer (resolution 2 cm^−1^).

### DFT calculations

Quantum-chemical calculations were used to obtain structural models of the rotaxane, and to support the interpretation of the T2DIR spectra based on empirical assignments. In the previous work^11^, we have used the B3LYP functional with the 6-31Gd basis set. The effect of the solvent acetonitrile was included by using the Polarizable Continuum Model. In the present work, we have explored the effects of using a larger basis set and different functionals on the structure and spectra for a representative conformation of the naphthalimide anion−macrocycle complex. In the model systems (Fig. 4), all features are included which we found to have an effect on the calculated frequencies of the vibrations of interest. Thus, it was found necessary to include a sufficiently long alkyl chain on the imide nitrogen, and use *t*-butyl groups on the naphthalimide ring. We have used a linear butyl chain (as we did previously^11^) but also a gauche-pentyl chain on the imide nitrogen, in order to test the effect of further symmetry lowering. We conclude from the calculated data (summarised in Supplementary Table 2 and discussed in the Supplementary Discussion) that the calculations with the B3LYP and B97D functionals using the 6-311G(d,p) basis set reproduce the experimental vibrational frequencies very well, but only slightly better than the B3LYP/6-31G(d) calculations. The results of the M062X/cc-pVDZ calculation are in substantially worse agreement with the experiment. We can link the computed and experimental frequencies in order to achieve the best possible agreement after simple linear scaling, with the condition that the computed IR intensity should be high (>100 km mol^−1^).

The coupling between the two CO-stretch modes in ni^•−^ was ~100 cm^−1^ (directly obtained from the difference between the symmetric and antisymmetric CO-stretch modes), which is much larger than the linewidth of the ni^•−^ CO-stretch IR peaks; hence in this case no localisation on single CO bonds occurs, and the ni^•−^ CO-stretch peak at ~1590 cm^−1^ originates the combined in-phase motion of the two ni^•−^ CO groups. Exactly the opposite is found for the amide I mode of the macrocycle: the calculations (regardless of the model used) show that the frequencies of the four possible combinations fall within 10 cm^−1^, which is much less than the experimentally observed linewidth, so that in this case the modes are localised on individual CO bonds.

### Data availability

The datasets generated and analysed during the current study are available from the corresponding authors on reasonable request.

## Electronic supplementary material

Supplementary Information
