# Supplementary material for: Transient two-dimensional vibrational spectroscopy of an operating molecular machine
Source: Nat Commun. 2017 Dec 20;8:2206. doi: 10.1038/s41467-017-02278-6 (PMC5738383; doi:10.1038/s41467-017-02278-6)
Supplement: Supplementary file 1 — Supplementary Information [file 41467_2017_2278_MOESM1_ESM.pdf]

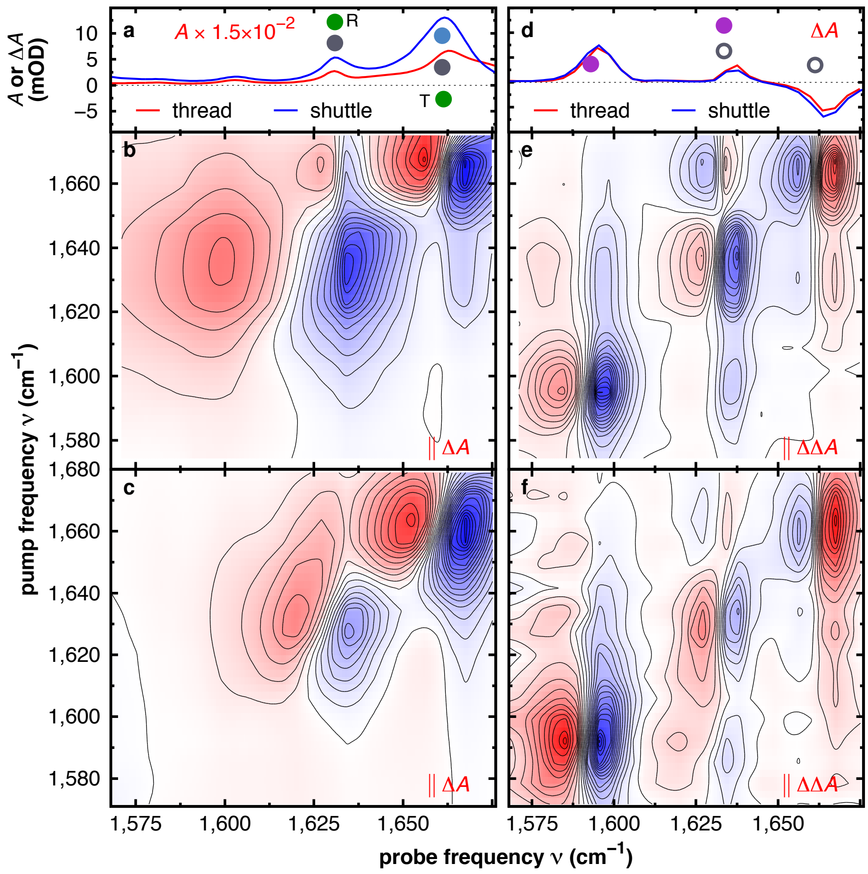

Supplementary Figure 1: **a** 1DIR spectrum of the bare thread and molecular shuttle. In the thread, the succ$_{HB}$ mode (green dot marked with T) is blue shifted with respect to the succ$_{HB}$ mode of the rotaxane (green dot marked with R). The thread also lacks the absorption bands associated with the macrocycle. **b** 2DIR spectrum (ΔA) of the thread. **c** 2DIR spectrum (ΔA) of the molecular shuttle. **d** T1DIR spectra (ΔA) of the thread and molecular shuttle at t_{UV} = 50 ns. **e** T2DIR spectrum ($\Delta\Delta A$) of the thread at t_{UV} = 50 ns. **f** T2DIR spectrum ($\Delta\Delta A$) of the molecular shuttle at t_{UV} = 50 ns. The 2DIR and T2DIR spectra are measured with a parallel (||) polarised IR-pump pulse with respect to the probe pulse. The coloured closed and open circles correspond to the labeled components of the molecular shuttle in Fig. 1a of the main text.

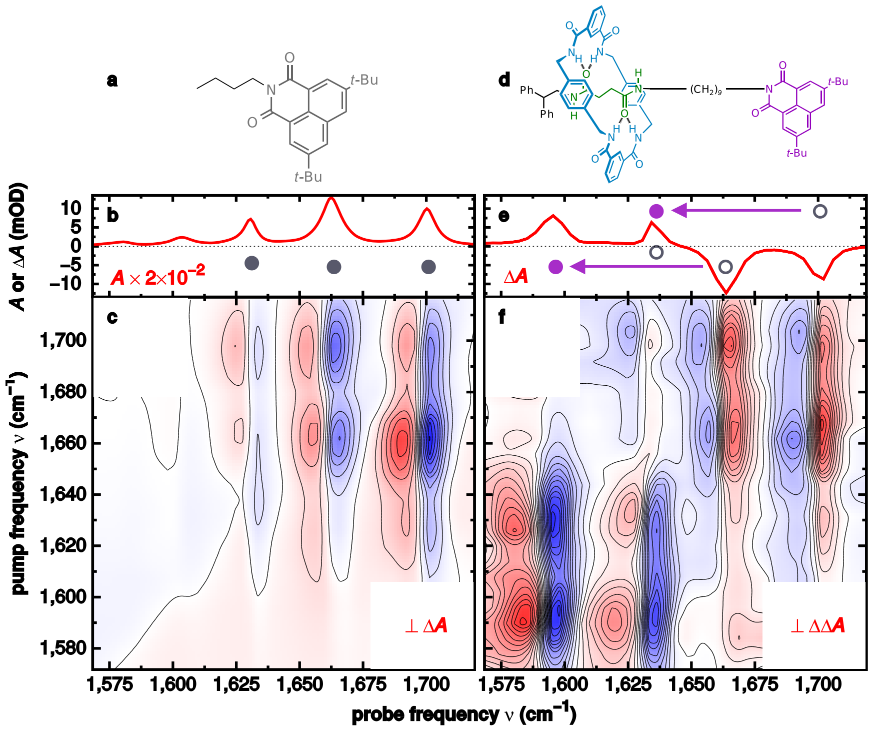

Supplementary Figure 2: Comparison between the ground state 2DIR spectrum of a model compound and the T2DIR spectrum of the molecular shuttle at 50 ns after UV excitation. **a** Chemical structure of the ni model compound. **b** 1DIR spectrum of the model compound. **c** 2DIR ($\perp$ ΔA) spectrum of the model compound. **d** Chemical structure of the molecular shuttle in the triplet state. **e** T1DIR spectrum (ΔA) of the molecular shuttle at t_{UV} = 50 ns. The arrows indicate the shift in frequency caused by the excitation of the ni station. The coloured closed and open circles labelling the spectral features correspond to the coloured components of the molecules. **f** T2DIR ($\perp$ $\Delta\Delta A$) spectrum of the molecular shuttle. The 2DIR and T2DIR spectra are measured with a pump pulse perpendicularly polarised with respect to the probe pulse.

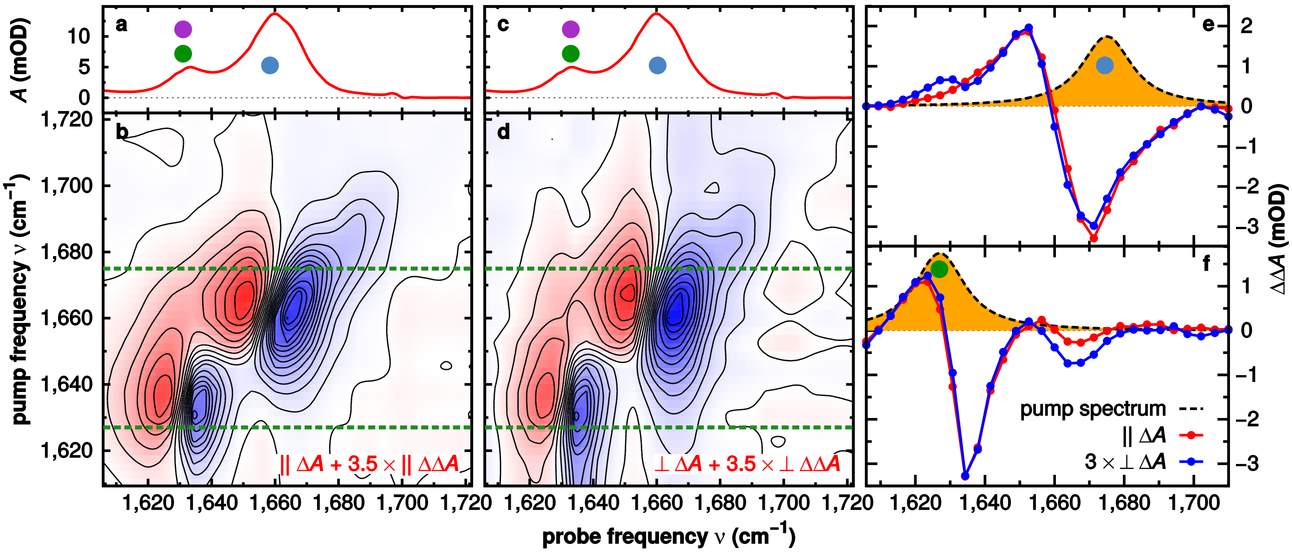

Supplementary Figure 3: The constructed ni-free 1DIR (**a**) and 2DIR spectra (**b**) of the molecular shuttle measured with a parallel (||) polarised IR-pump pulse with respect to the probe pulse. The constructed ni-free 1DIR (**c**) and 2DIR spectra (**d**) of the molecular shuttle measured with a perpendicularly (⊥) polarised IR-pump pulse with respect to the probe pulse. The dashed green lines denote the pump positions of the slices in **e** and **f**. The 2DIR spectra are measured with a perpendicularly (⊥) polarised IR-pump pulse with respect to the probe pulse **e** Cross section of the ni station-free 2DIR spectrum (ΔA), where the yellow peak denotes the resonant excitation at 1,675 cm^{-1}. **f** Cross section of the ni station-free 2DIR spectrum (ΔA), where the yellow peak denotes the resonant excitation at 1,627 cm^{-1}. In the slices, the red curve is the spectrum measured with a parallel (||) polarised IR-pump pulse, and the blue curve is the spectrum measured with a perpendicularly (⊥) polarised IR-pump pulse with respect to the probe pulse. The perpendicular signal in both T2DIR cross sections is multiplied by a factor of three for an easy comparison with the parallel signal. The coloured closed and open circles correspond to the labeled components of the molecular shuttle in Fig. 2 of the main text.

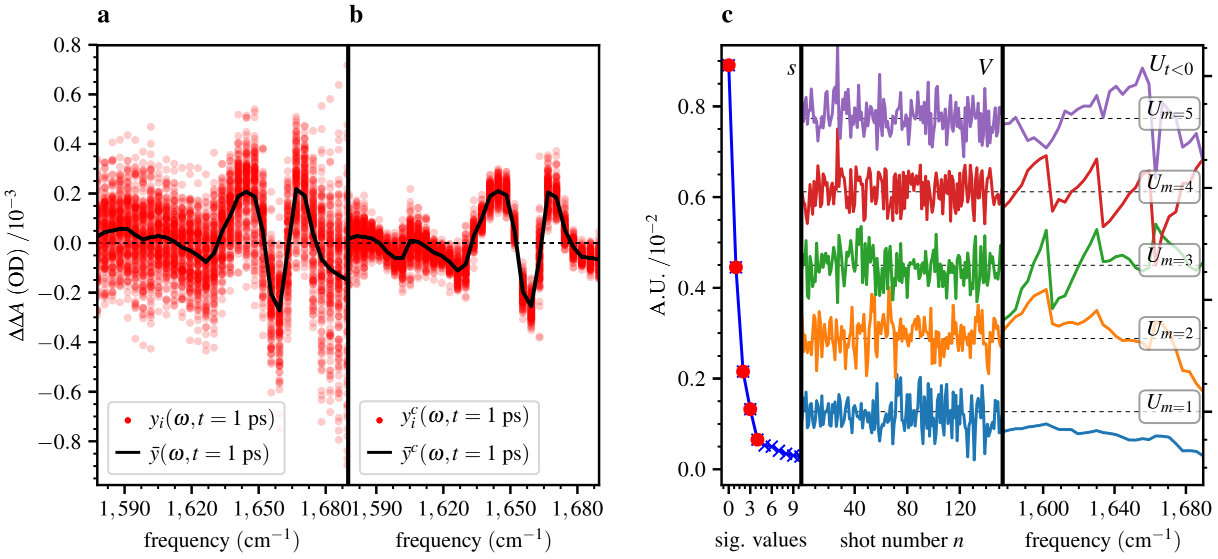

Supplementary Figure 4: Example of the SVD-based reduction of uncertainty using the perpendicularly polarized T2DIR signal ν_{pump} = 1,654 cm^{-1}. **a** The n measured points $y(\omega, t = 1 \text{ ps})$ and their mean $\bar{y}(\omega, t = 1 \text{ ps})$. **b** Subtraction of the two most significant components ($U_{t<0}$, panel **c**) from $y(\omega, t = 1 \text{ ps})$ and the new mean. **c** Singular values (5 most significant marked in red, s), target (V)- and projection ($U_{t<0}$)- vectors of points $y(\omega, t < 0)$.

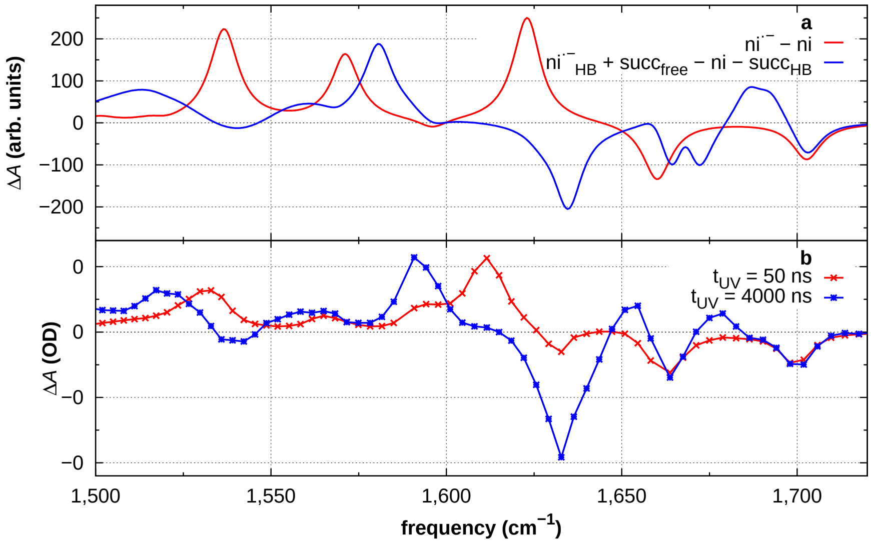

Supplementary Figure 5: Calculated (**a**) and experimental (**b**) transient 1D-IR spectra. The calculations were performed on the following model compounds: ni = ni$^{\bullet-}$ = n-propylnaphthalimide; $\text{ni}_{\text{HB}}^{\bullet-}$, succ_{HB}, and $\text{succ}_{\text{free}}$, at the B3LYP/6-31G* level. See Fig. 4 of the main text for the structures of the model compounds.

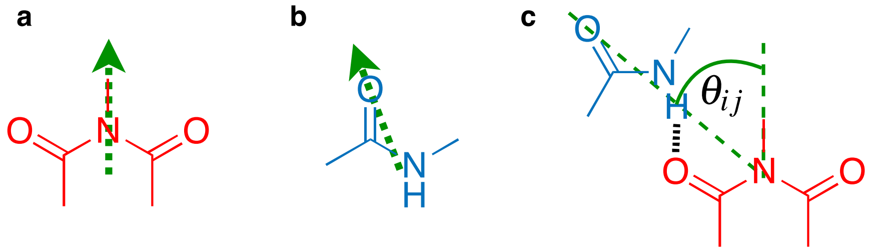

Supplementary Figure 6: **a** Fragment of the $ni^{\bullet-}$ station containing the CO groups. The dashed green arrow denotes the transition dipole moment of the $ni^{\bullet-}_{HB}$ mode. **b** Fragment of the macrocycle containing one amide group. The dashed green arrow denotes the transition dipole moment of the $mc_{HB\cdots ni^{\bullet-}}$ mode. **c** Schematic representation of the switched conformation of the molecular shuttle. The dashed lines lie along the normal modes of the individual components, the angle between them is θ_{ij}.

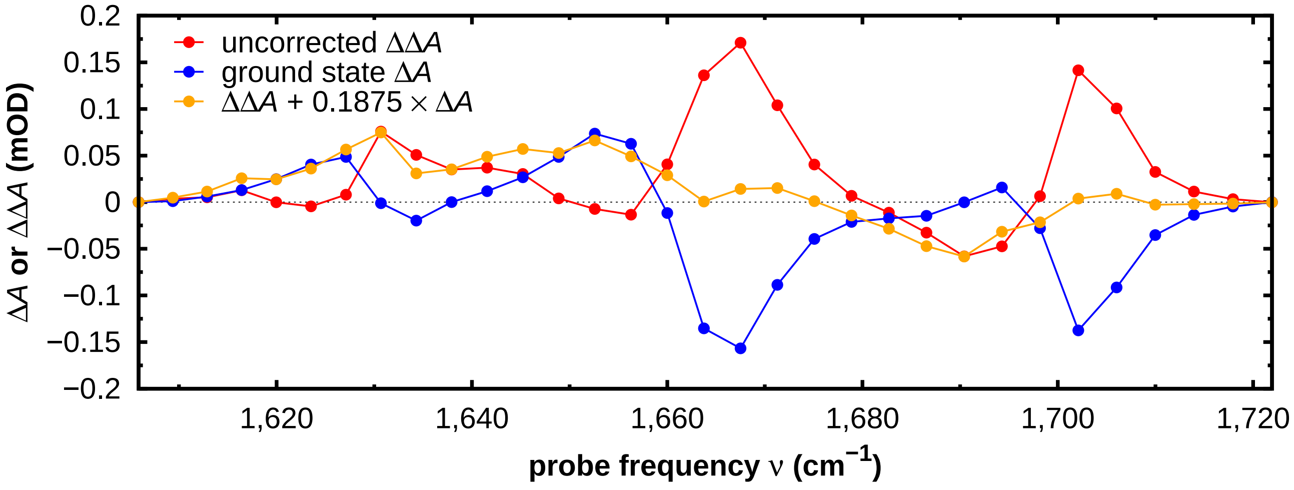

Supplementary Figure 7: The ΔA and $\Delta\Delta A$ response of the molecular shuttle at t_{UV} = 1,000 ns when the ni_s mode is pumped. The spectra are measured with a perpendicularly (⊥) polarised IR-pump pulse with respect to the probe pulse.

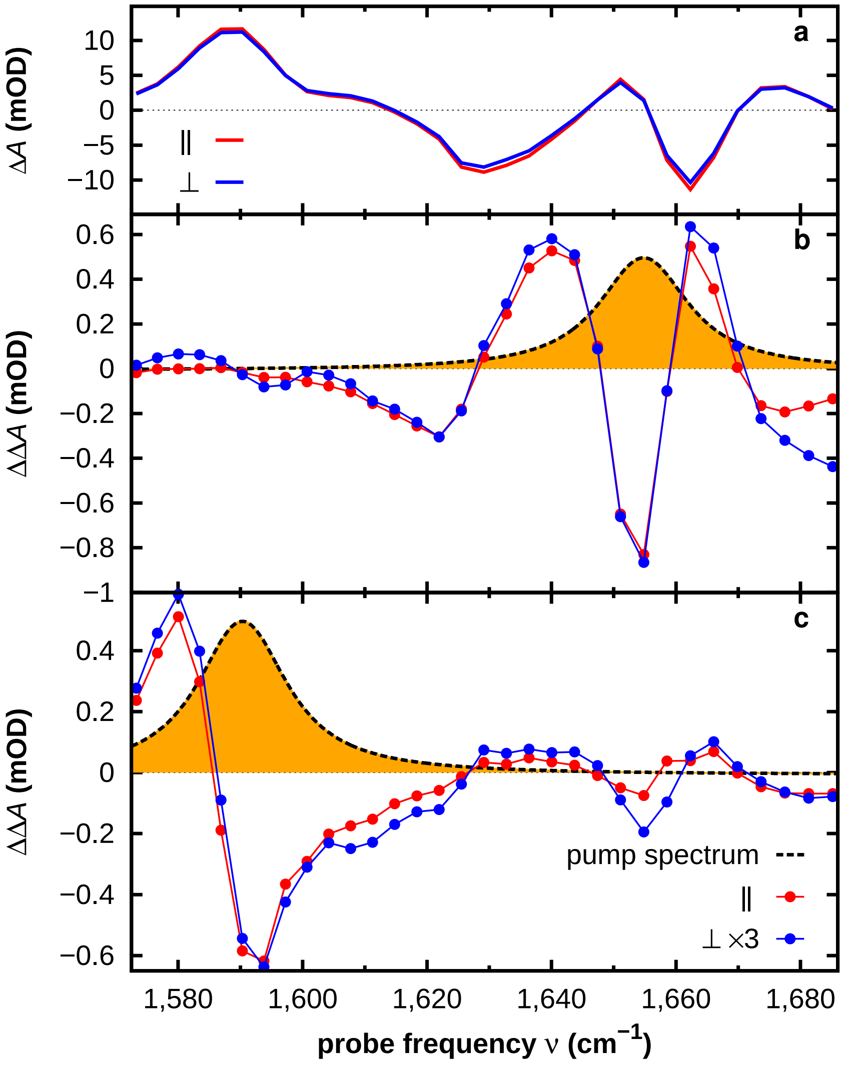

Supplementary Figure 8: **a** T1DIR spectrum (ΔA) of the molecular shuttle 1,000 ns after UV excitation. The colored closed and open circles correspond to the labeled components of the molecular shuttle in main text Fig. 2. **b** T2DIR spectrum cross section ($\Delta\Delta A$), where the yellow peak denotes the resonant excitation at 1,654 cm^{-1}. **c** T2DIR spectrum cross section ($\Delta\Delta A$), where the yellow peak denotes the resonant excitation at 1,592 cm^{-1}. In all spectra, the red curve is the spectrum measured with a parallel (‖) polarised IR-pump pulse, and the blue curve is the spectrum measured with a perpendicularly (⊥) polarised IR-pump pulse with respect to the probe pulse. The perpendicular signal in both T2DIR cross sections is multiplied by a factor of three to facilitate comparison with the parallel signal.

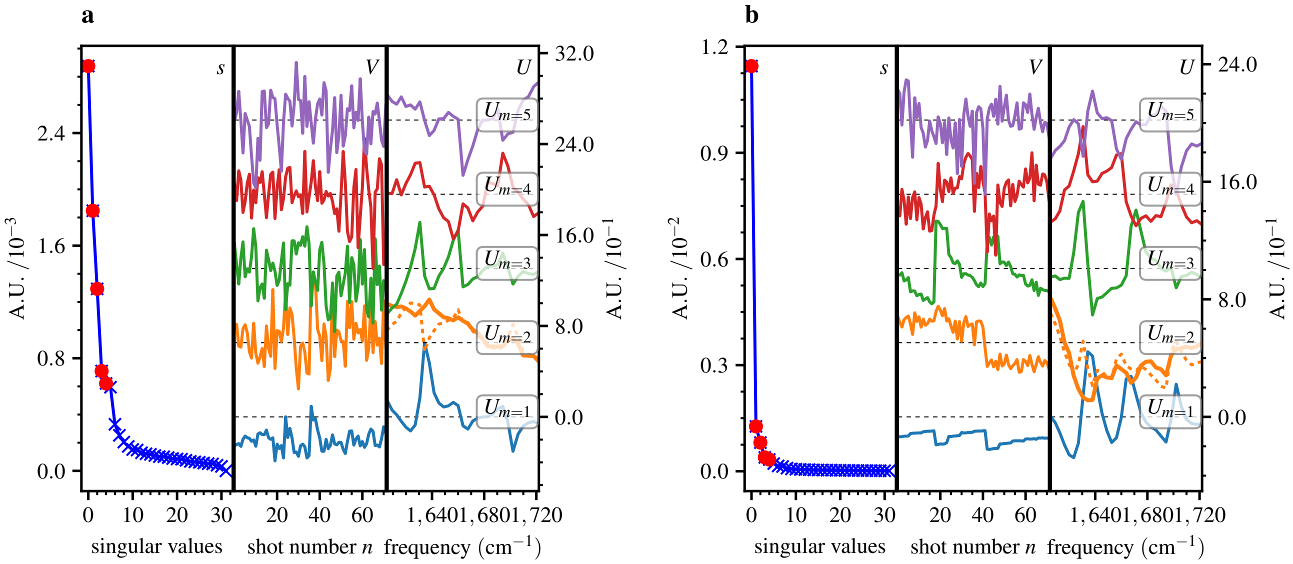

Supplementary Figure 9: Singular values (5 most significant marked in red, s), target (V)- and projection (U)- vectors of perpendicularly polarised ν_{pump} = 1,627 cm^{-1}, $y(\omega,t) = T_{\mathrm{UV,IR}}$ (**a**), and $y(\omega,t) = T_{\mathrm{IR}}$ (**b**). The dotted lines represent the uncorrected $U_{m=2}$ projection vector.

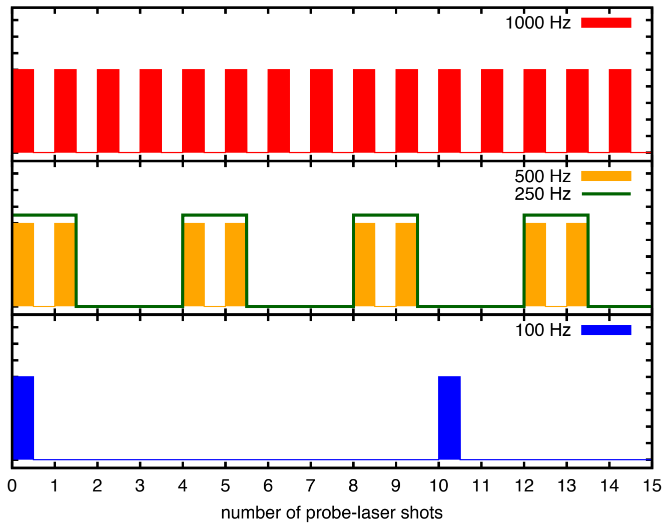

Supplementary Figure 10: Electronic timing of a pump-pump-probe experiment. The coloured bars represent the probe (red), pump (orange), and UV (blue) laser pulses. The green line shows the electronic pump present signal which encompasses two subsequent pump pulses.

Supplementary Table 1: Summary of the measured and calculated absorption bands. The vibrational modes with their corresponding frequencies and description of the molecular shuttle in the ground, triplet, and radical-anion state. s and as stand for the symmetric and antisymmetric CO stretch vibrations of the naphthalimide station in the electronic ground state, respectively; Ar stands for aromatic ring vibration; rad. anion for radical anion. The calculated frequencies were obtained from DFT at the B3LYP/6-31G(d) level (scaling factor 0.973) on the following model compounds: [a] *n*–propyl–naphthalimide station; [aT] *n*–propyl–naphthalimide station in the triplet state; [a•−] radical anion *n*–propyl–naphthalimide station; [b] methyl–succinamide station–macrocycle pseudo–rotaxane; [c] radical anion *n*–propyl–naphthalimide station–macrocycle pseudo–rotaxane; [d] methyl–succinamide station.

Mode	exp. frequency (cm^{-1})	calculated and scaled	electronic state of ni	colour code
ni_{s}	1,700	1,703 [a]	ground	●
ni_{as}	1,663	1,660 [a]	ground	●
$mc_{HB\cdots succ}$	1,663	1,664–1,673 [b]	ground	●
ni_{Ar}	1,632	1,627 [a]	ground	●
$succ_{HB}$	1,632	1,627 [b]	ground	●
ni_{Ar}	1,603	1,596 [a]	ground	●
ni^{*}_{s}	1,632	1,629 [aT]	triplet	●
ni^{*}_{as}	1,591	1,584 [aT]	triplet	●
$ni^{\bullet-}_{free}$	1,613	1,623 [a•−]	rad. anion	●
$succ_{free}$	1,678	1,689 [d]	rad. anion	●
$mc_{HB\cdots ni^{\bullet-}}$	1,654	1,658–1,666 [c]	rad. anion	●
$ni^{\bullet-}_{HB}$	1,592	1,579 [c]	rad. anion	●

Supplementary Table 2: Calculated normal-mode frequencies (scaling factor 0.973) relevant to the 2DIR experiments of the model system shown in Fig. 4 of the main text. All numbers are in cm^{-1}; multiple numbers are given for the computed vibrations because both n-butyl and n-pentyl chains were included, and because in many cases several modes contribute to the observed bands.

Experimental	approximate description	B3LYP/ 6–31G(d)	B3LYP/ 6–311G(d,p)	B97D/ 6–311G(d,p)	M062X/ CC–pVDZ
1,488	ni CO asymmetric	1,486	1,482	1,492	1,488–1,492
1,515–19	ring/ni CO asymmetric	1,513–1,517	1,515–1,520	1,523–1,524	1,504–1,510
1,556	amide II	1,552–1,559	1,554–1,563	1,554–1,562	1,521–1,561
1,581	ni CO symmetric	1,579	1,580	1,587–1,588	1,581–1,585
1,660–68	macrocycle CO	1,658–1,666	1,653–1,662	1,645–1,655	1,666–1,683
largest absolute deviation		13	12	9	34

Supplementary Methods

The T2DIR setup

For the T2DIR pump-probe experiments we use a commercial Ti:sapphire laser (Coherent Legend Elite, 3.5 mJ, 35 fs FWHM) synchronized with a commercial, pulsed Nd:YAG laser of which we use the third harmonic (IB Laser DiNY pQ, 355 nm, 0.58 mJ, 3.6 ns). A variable, computer-controlled time delay between the two laser outputs is achieved in a similar manner as reported by other groups[1,2]. A signal generated from the Ti:sapphire oscillator (80 MHz) output is amplified and frequency-divided to produce a 1 kHz signal. This signal triggers a pulse generator which provides the triggering for the YLF pump laser and Pockels-cell driver of the regenerative amplifier, an electronically gated amplifier used to record the signals of the MCT-detector array, and a computer-controlled electronic delay generator (Berkeley Nucleonics Corporation Model 575-4C). The latter provides the triggering for the Nd:YAG laser that generates the UV pump pulse. The maximum delay between the UV and mid-IR laser pulses is determined by the repetition rate of the Ti:sapphire laser (1 ms).

The 355 nm pulse length is determined from a cross-correlation with the mid-IR pulses obtained by differentiating the pump-probe signal in a Ge plate. From a fit to a gaussian function we obtain a FWHM of 3.6$\pm$0.4 ns for the pump pulse. The Nd:YAG laser is pumped at 500 Hz and Q-switched at 100 Hz. Using the amplified 800 nm output of the Legend and an optical setup described elsewhere[3] we obtain mid-IR pulses with a duration of $\sim$150 fs, a bandwidth of 200 cm^{-1} and an energy of 20 μJ. Probe and reference pulses are obtained from the mid-IR light by reflection off the front and back surfaces of a wedged BaF_2 window. The remainder is passed through an IR Fabry-Perot interferometer, resulting in pump pulses with a bandwidth of 25 cm^{-1}. The centre frequency of the light is varied by adjusting the distance between the parallel mirrors of the IR Fabry-Perot interferometer using a feedback-controlled piezoelectric mount. The polarisation of the IR pump pulse is set at 45° with respect to that of the probe pulse using a MgF_2 zero-order λ-half plate. Subsequently, the polarisation of the measured spectrum is selected using a polariser situated directly after the sample set at either 0° (parallel spectrum) or 90° (perpendicular spectrum) with respect to the pump polarisation. The pump pulses have an intensity envelope that is approximately single-sided exponential with a FWHM of 800 fs, as determined from a cross correlation measured by using two-photon absorption in InAs placed in a sample cell similar to the one used in the T2DIR experiment.

The T2DIR experiment is performed by focusing the 355 nm output 3 cm before the sample with an f = 300 mm CaF_2 lens. The changes induced in the sample are monitored by the two mid-IR probe pulses, which are spatially overlapped with the pump beam, at various time delays between the UV-pump and mid-IR pulses. The mid-IR reference beam passes through an area of the sample not influenced by the pump. The mid-IR pump, probe and reference beams are focused through the sample by means of an f = 100 mm off-axis parabolic mirror. At the sample, the UV and mid-IR beam diameters are 1 mm and 200 μm, respectively. The polarisation of the UV-pump and mid-IR probe are perpendicular with respect to one another. Transient absorption changes are measured by frequency-dispersed detection of the mid-IR pulses using a 2 $\times$ 32 HgCdTe (MCT) array detector (Infrared Associates).

The T2DIR experiment

The T2DIR spectrum, $\Delta\Delta A\left(\omega_1, \omega_2, t_{\mathrm{UV}}, t_{\mathrm{IR}}\right)$, is the difference between the 2DIR spectrum of the electronic excited state species, $\Delta A_{UV,IR}\left(\omega_1, \omega_2, t_{\mathrm{UV}}, t_{\mathrm{IR}}\right)$, and the 2DIR spectrum of the electronic

ground state species, $\Delta A_{IR}(\omega_1,\omega_2,t_{\mathrm{IR}})$:

$$\Delta\Delta A(\omega_1,\omega_2,t_{\mathrm{UV}},t_{\mathrm{IR}}) = \Delta A_{UV,IR}(\omega_1,\omega_2,t_{\mathrm{UV}},t_{\mathrm{IR}}) - \Delta A_{IR}(\omega_1,\omega_2,t_{\mathrm{IR}}) \tag{1}$$

$$\Delta\Delta A(\omega_1,\omega_2,t_{\mathrm{UV}},t_{\mathrm{IR}}) = A_{UV,IR}(\omega_1,\omega_2,t_{\mathrm{UV}},t_{\mathrm{IR}}) - A_{UV}(\omega_1,\omega_2,t_{\mathrm{UV}}) - A_{IR}(\omega_1,\omega_2,t_{\mathrm{IR}}) - A_0(\omega_1,\omega_2)\ . \tag{2}$$

$A_0(\omega_1,\omega_2)$ is the absorption of the sample when neither pump pulse is present. The signals are measured in terms of transmission with $A = -\log_{10}(T/T_0)$. This yields the following:

$$\begin{aligned}\Delta\Delta A(\omega_1,\omega_2,t_{\mathrm{UV}},t_{\mathrm{IR}}) &= \\ -\left[a\cdot\log_{10}\left(\frac{T_{\mathrm{UV,IR}}(\omega_1,\omega_2,t_{\mathrm{UV}},t_{\mathrm{IR}})}{T_{\mathrm{UV}}(\omega_2,t_{\mathrm{UV}})}\right) + (1-a)\cdot\log_{10}\left(\frac{T_{\mathrm{IR}}(\omega_1,\omega_2,t_{\mathrm{UV}},t_{\mathrm{IR}})}{T_0(\omega_2)}\right)\right] \\ +\log_{10}\left(\frac{T_{\mathrm{IR}}(\omega_1,\omega_2,t_{\mathrm{IR}})}{T_0(\omega_2)}\right)\ ,\end{aligned} \tag{3}$$

where T_0 is the transmission of the sample in the absence of a pump pulse, a is the fraction of molecules excited by the UV pump, ω_1 the pump frequencies, ω_2 the probe frequencies, t_{UV} the delay between the UV-pump and 2DIR pulse pair, and t_{IR} the delay between the IR-pump and IR-probe pulses. The terms in the square brackets are observed at the same time by the probe when both UV and IR pump pulses are acting on the sample.

We use the timing scheme shown in Supplementary Fig. 10 to obtain the signals necessary to construct $\Delta\Delta A$ as shown in equation 3: the sample is probed at a repetition rate of 1 kHz. The mid-IR pump pulse is optically chopped at a frequency of 250 Hz such that two sequential pulses are allowed to pass and the following two sequential pulses are blocked, effectively pumping the sample at 500 Hz. The UV laser is run at a repetition rate which is not a divider of 250 Hz (we choose 100 Hz). In this manner, the following absorption spectra required for the generation of the T2DIR spectrum can be recorded: the ground state absorption, A_0, is measured at each laser shot where both UV and IR pumps are off (occurring at 450 Hz). The vibrationally excited state absorption, A_{IR} at a certain pump frequency and delay time, is measured at each shot where only the IR pump pulse acts on the sample (occurring at 450 Hz). The electronic excited state absorption, A_{UV} at a certain delay time, is measured at each shot where only the UV pump pulse acts on the sample (occurring at 50 Hz). The vibrationally excited absorption of the electronic excited state species, $A_{\mathrm{UV,IR}}$ at a certain IR-pump frequency and delay time, is measured at each shot where both IR and UV pumps act on the sample (this occurs at 50 Hz).

Baseline correction

The absorption of the sample y observed with a certain pixel (corresponding to a frequency ω), at a certain delay (t) is determined by the mean $\bar{y}(\omega,t)$ of all individual shots y_i, and the corresponding error is determined by the standard deviation $\sigma(\omega,t)$ of these points:

$$\bar{y}(\omega,t) = \frac{1}{n}\sum_{i=1}^{n} y_i(\omega,t), \quad \sigma(\omega,t) = \sqrt{\frac{1}{n}\sum_{i=1}^{n}\left(y_i(\omega,t)-\bar{y}(\omega,t)\right)^2}, \quad y = \left\{T_0, T_{\mathrm{IR}}, T_{\mathrm{UV}}, T_{\mathrm{UV,IR}}\right\} \tag{4}$$

where n is the total number of repeats of the measurement. The T2DIR measurements contain random baseline fluctuations which can be averaged out by performing a sufficient number measurements, thereby revealing the T2DIR signal (see Supplementary Fig. 4a).

In order to reduce the spread of the measurements caused by stochastic baseline fluctuations, we employed a singular-value-decomposition (SVD) based method developed by Haldrup[4]. Briefly, an SVD is performed on the measurements before t_{UV} = 0, which yields the projection vectors, singular values, and target vectors (U, s, and V^T respectively) of the baseline fluctuations (see Supplementary Fig. 4c). Next, the contribution of each baseline component $a_i(\omega, t < 0)$ to each measurement $y_i(\omega, t)$ is obtained from a least-squares fit of the target vectors $U_{t<0}$ to the measurements at delay points $t > 0$, $y(\omega, t > 0)$. We subtract the most significant of the components $\tilde{U}_{t<0}$ from the dataset, thereby reducing the spread of the individual measurements (Supplementary Fig. 4b):

$$y_i^c(\omega, t > 0) = y_i(\omega, t > 0) - \tilde{a}_i(\omega, t < 0) \cdot \tilde{U}_{t<0} \tag{5}$$

Subsequently, we employ robust statistics to further reduce the uncertainty of the measured points[5]. As explained in Supplementary Equation 4: we take the mean of the data $\tilde{y}(\omega, t)$ to determine the value of y, and the uncertainty is determined via the median absolute deviation (MAD) of the spread of the data points $y_i(\omega, t)$:

$$\tilde{y}(\omega, t) = \text{median}\left(y_i(\omega, t)\right), \quad \text{MAD} = \text{median}\left(\left|y_i(\omega, t) - \tilde{y}(\omega, t)\right|\right), \quad y = \left\{T_0, T_{\mathrm{IR}}, T_{\mathrm{UV}}, T_{\mathrm{UV,IR}}\right\} . \tag{6}$$

The MAD is converted to a standard deviation using the relationship $\sigma = 1.4826 \cdot \text{MAD}$.

In the case of the initial state of the molecular shuttle (see main text Fig. 1a), we did not record data points at delays $t < 0$. The 1st and 2nd projection vectors can therefore manifest mixing of both signal and baseline artifacts (see Supplementary Fig. 9) which is especially the case with the T2DIR signals. As a consequence, we would severely distort our data if we naively applied the SVD-based baseline reduction method as described above. We therefore remove the signal contribution (1st projection vector) from the 2nd projection vector. The 1st component of the $U_{t<0}$ of the final state T2DIR signal in Supplementary Fig. 4c is predominantly a featureless baseline artefact compared to the T2DIR signal. For the following analysis, we use the fact that the 2nd component of the initial state T2DIR should be of a similar nature to the 1st component of the $U_{t<0}$ of the final state and that the 1st component of the initial state predominantly contains signal. The 2nd derivative with respect to frequency is a measure with which to evaluate only the structured part of the 2nd component, and ignore featureless baseline artefacts. To remove structured features from the 2nd component $U_{m=2}$ (where m specifies a component), we first take the 2nd derivative of all the components with respect to frequency $U'' = \frac{d^2U}{d\omega^2}$. The magnitude $b_{m\neq2}$ of the projection vectors $U_{m\neq2}$ in $U_{m=2}$ is determined by a least-squares fit of the first four $U''_{m\neq2}$ to $U''_{m=2}$. The corrected 2nd projection vector $U^c_{m=2}$ is obtained as follows:

$$U^c_{m=2} = U_{m=2} - \sum_{m=1}^{k} b_{m\neq2} \cdot U_{m\neq2} , \tag{7}$$

where, in our case, $k = 5$. The result is shown in Supplementary Fig. 9. We subsequently continue with the SVD-based baseline removal as described above.

The baseline fluctuation target vectors $V_{m\neq1}$ are randomly distributed about 0, whereas the signal component target vector $V_{m=1}$ is predominantly either positive or negative. This is probably caused by laser-power fluctuations or changing pump-probe beam overlap due to ambient temperature fluctuations of over the course of hours. A final reduction in the spread of $y_i(\omega, t)$ is achieved by reconstituting $y_i(\omega, t)$ with the normalised 1st projection vector $U_{N_{m=1}}$ to the median of the corresponding target vector $V_{m=1}$ as follows:

$$y_i^c(\omega, t) = U_{N_{m=1}} \cdot s \cdot V \tag{8}$$

Materials

All experiments on the molecular shuttle were carried out on a solution of the rotaxane (5×10^{-4} M) and 1,4-diazabicyclo[2.2.2]octane (DABCO, 5×10^{-2} M) in CD_3CN (Eurisotop, $>$99.8% D purity). Neither the solvent nor DABCO has absorption bands in the spectral region of interest. Argon was bubbled through the sample for $>$15 min in order to remove dissolved oxygen from the solution. The sample was kept in a thermostatted sealed IR cell consisting of two CaF_2 windows separated by a 5 mm spacer. The sample cell was constantly moved in a Lissajous pattern to avoid residual heating and photochemical degradation of the sample. All steady-state Fourier-transform infrared (FTIR) spectra were measured on a Bruker Vertex 70 spectrometer (resolution 2 cm^{-1}).

Supplementary Note 1: Comparison between the T2DIR spectrum of the molecular shuttle and bare thread at t_{UV} = 50 ns

The T2DIR spectra of the molecular shuttle and the bare axle (the thread) at t_{UV} = 50 ns are shown in Supplementary Fig. 1. The 1DIR spectrum of the rotaxane (Supplementary Fig. 1a) is discussed in the main text. The thread lacks the contribution of the amide I modes of the macrocycle at 1,663 cm^{-1}. The absence of the macrocycle also results in a blue shift of the amide I modes of the succ station from 1,632 cm^{-1} to 1,678 cm^{-1}. These spectral differences are also visible in the 2DIR spectra of the thread and the shuttle (Supplementary Fig. 1b and c). In the 2DIR spectra of the thread, the cross peak between the ni$_{as}$ and ni$_{Ar}$ modes at (1,663 cm^{-1}, 1,632 cm^{-1}) is clearly observed. In the case of the shuttle, the v = 1→2 transition of the diagonal peak of the mc$_{HB\cdots succ}$ mode obscures the negative signal of the ni$_{as}$/ ni$_{Ar}$ cross peak, which is the reason we only observe a positive signal at ν_{pump} = 1,625 cm^{-1}. In the thread, the v = 0→1 transition of the ni$_{Ar}$ mode at 1,632 cm^{-1} overlaps with the positive signal of the cross peak between the ni$_{Ar}$ mode at 1,632 cm^{-1} and the ni$_{Ar}$ mode at 1,605 cm^{-1}. In the spectrum of the shuttle the diagonal peak of the succ$_{HB}$ mode obscures the ni$_{Ar}$ modes at 1,632 cm^{-1} and 1,605 cm^{-1}. The T1DIR spectra of the thread and molecular shuttle are the same at t_{UV} = 50 ns (peaks are discussed in the main text and in Supplementary Note 2).

The T2DIR spectra of the thread and shuttle are shown in Supplementary Fig. 1e and f, respectively. The peaks in both spectra are discussed in Supplementary Note 2. The spectra match on-to-one because only the triplet species is observed at this t_{UV}-delay and the depletion only involves to the ni station (Supplementary Note 2). Also, the similarity between the thread and rotaxane means that the macrocycle has not yet left the succ station.

Supplementary Note 2: Selective depletion of the naphthalimide station.

The selective depletion is supported by Supplementary Fig. 2 in which we show the 1DIR and 2DIR spectrum of a model compound comprising only the ni station. The 2DIR spectrum of the model compound Supplementary Fig. 2c corresponds one-to-(minus)one with the ni depletion spectrum of the ni* T2DIR spectrum. The three diagonal peaks in this spectrum are the ni_s (ν_{pump}, ν_{probe}) = (1,700 cm^{-1}, 1,700 cm^{-1}), ni_{as} (1,663 cm^{-1}, 1,663 cm^{-1}), and ni_{Ar} (1,632 cm^{-1}, 1,632 cm^{-1}) modes. The prominent cross peaks found at (1,700 cm^{-1}, 1,663 cm^{-1}), (1,700 cm^{-1}, 1,632 cm^{-1}), (1,663 cm^{-1}, 1,632 cm^{-1}), (1,663 cm^{-1}, 1,700 cm^{-1}), (1,632 cm^{-1}, 1,700 cm^{-1}), and (1,632 cm^{-1}, 1,663 cm^{-1}) indicate strong coupling between all the ni modes. The coupling between the ni_s and ni_{as} (at (1,700 cm^{-1}, 1,663 cm^{-1}) and (1,632 cm^{-1}, 1,700 cm^{-1})) is particularly intense because these modes involve the same two CO groups[6]. In addition to the inverted ni spectrum, the ni* T2DIR spectrum (Supplementary Fig. 2f) contains two diagonal peaks at (1,632 cm^{-1}, 1,632 cm^{-1}) and (1,592 cm^{-1}, 1,592 cm^{-1}) which belong to the ni^*_s and ni^*_{as} modes, respectively. Analogous to the ground state, the cross peaks between the ni^*_s and ni^*_{as} modes at (1,632 cm^{-1}, 1,592 cm^{-1}) and (1,592 cm^{-1}, 1,632 cm^{-1}) are intense, indicating strong coupling. The ni^*_s mode completely cancels the inverted diagonal signal of the 1,632 cm^{-1} ni_{Ar} band. However, the presence of the latter is evident from the depletion cross peaks at (1,700 cm^{-1}, 1,632 cm^{-1}), (1,663 cm^{-1}, 1,632 cm^{-1}), (1,632 cm^{-1}, 1,700 cm^{-1}), and (1,632 cm^{-1}, 1,663 cm^{-1}). The fact that the ni^*_s mode coincides with the aromatic ring-stretch mode can be inferred from the comparison of the 2DIR spectrum of the model compound with the T2DIR spectrum of the rotaxane. Importantly, it demonstrates that the selective depletion of the ground state can uncover hidden spectral features in systems where comparison with model systems is not possible.

Supplementary Note 3: Estimating the rotational diffusion correlation time of a fully extended rotaxane

Assuming the rotaxane has a spherical shape, the Stokes-Einstein-Debye equation for rotational diffusion[7]:

$$\theta = \frac{\eta \cdot V}{k_B \cdot T} \,, \tag{9}$$

where θ is the rotational correlation time of the system (s), η is the effective microscopic viscosity (10^{-3}·kg m^{-1} s^{-1}), V is the effective volume of the solvated system (m^3), k_B is the Boltzmann factor (J · K^{-1} = kg m^2 s^{-2} K^{-1}), T is the temperature (K). Substituting: η = 3.4×10^{-4} kg m^{-1} s^{-1}, (obtained from Horng *et.al.*[8]), $V = \frac{4}{3}\pi r^3$, r = 2.3 nm (thread fully extended[9]), T = 343 K^{-1} (the measurements are performed at 343 K).

$$\theta = \frac{3.4 \times 10^{-4} \cdot 6.37 \times 10^{-27}}{1.3806488 \times 10^{-23} \cdot 343} = 457 \text{ ps} \tag{10}$$

Supplementary Note 4: Cross sections of the naphthalimide station-free 2DIR spectrum of the molecular shuttle

The ground-state 1DIR spectrum of the molecular shuttle is shown in main text Fig. 3b. In addition to the peaks discussed previously, a peak is observed at 1,700 cm^{-1} which belongs to the symmetric CO-stretch vibration of the ni station (ni$_s$). The ni$_s$ mode is spectrally isolated and we can therefore use it as a measure for the amount of ni present in the spectrum. The corresponding 2DIR spectrum is shown in main text Fig. 3c. The diagonal absorption of the ni$_s$ mode is observed at (1,700 cm^{-1}, 1,700 cm^{-1}). Cross peaks between the ni$_s$ and the ni$_{as}$ and ni$_{Ar}$ modes are clearly visible at (1,700 cm^{-1}, 1,663 cm^{-1}), (1,700 cm^{-1}, 1,632 cm^{-1}), (1,663 cm^{-1}, 1,700 cm^{-1}), and (1,632 cm^{-1}, 1,700 cm^{-1}). The remaining features are discussed in the main text. As stated in the main text, the ground-state 2DIR spectrum of the shuttle contains overlapping absorptions of the ni station with both the macrocycle and succ station which makes direct conformational determination impossible. Supplementary Fig. 3 shows the ni-free 2DIR spectrum of the molecular shuttle from which we obtained the angle θ_{ij} between the mc$_{HB\cdots succ}$ and succ$_{HB}$ modes. In order to obtain θ_{ij}, we need to eliminate the contribution from the ni station. The 1DIR ni-free spectrum (Supplementary Fig. 3a and c) is constructed by subtracting the 1DIR spectrum of the ni station (Supplementary Fig. 2b) from that of the molecular shuttle. The spectra are normalised on the ni$_s$ mode before subtraction. The remaining peaks are the mc$_{HB\cdots succ}$ (1,663 cm^{-1}) and the succ$_{HB}$ (1,632 cm^{-1}) modes.

The ni-free 2DIR spectra (Supplementary Fig. 3b and d) are generated in a similar fashion as the 1DIR spectra, with the added benefit that we do not need record a separate ni station spectrum. The T2DIR spectrum of the triplet species contains the ground-state 2DIR depletion-spectrum of the ni station. By comparing intensities at 1,700 cm^{-1} we determine that in order to eliminate the contribution of the ni station, one needs to add the depletion spectrum multiplied by a factor of 3.5 to the ground-state 2DIR spectrum. The result for both pump polarisations is shown in Supplementary Fig. 3. The peaks observed in the 2DIR spectra have been discussed in the main text.

Supplementary Fig. 3e and f show cross-sections of the ni-free 2DIR spectra at the pump frequencies 1,675 cm^{-1} and 1,627 cm^{-1} respectively, indicated by the green dashed lines. The macrocycle and succ station absorptions overlap moderately. For the determination of the angle θ_{ij} between the vibrational modes, we choose to use cross-sections where the central frequency of the pump is slightly off-resonance. In this manner, we avoid direct excitation of both modes. When pumping at 1,675 cm^{-1} (Supplementary Fig. 3e), we observe a large resonant feature with v = 0→1 = 1,668 cm^{-1} and v = 1→2 = 1,654 cm^{-1}. A polarisation-dependent ΔA feature is observed on the flank of the v = 1→2 transition at 1,627 cm^{-1}. This is the positive part of the cross-peak, indicative of coupling between the macrocycle and the succinamide station. The cross-section of the ni-free 2DIR spectrum with ν_{pump} = 1,627 cm^{-1} shows (Supplementary Fig. 3f) a diagonal peak with v = 0→1 = 1,654 cm^{-1} and v = 1→2 = 1,623 cm^{-1}. A negative polarisation-dependent off-diagonal feature is observed at 1,668 cm^{-1}, which corresponds to the v = 0→1 transition of the macrocycle amide I mode. This is the complementary cross peak which indicates coupling between the succ$_{HB}$ and mc$_{HB\cdots succ}$ modes.

We determined the cross-peak anisotropy in the same fashion as was done for the switched state. The maximum change in absorption of the cross peaks were used for the values of $\Delta A_{\parallel}$ and $\Delta A_{\perp}$. Using equation 2 of the main text, we obtain two independent determinations of θ_{ij}, one from each off-diagonal feature, which allows us to estimate the uncertainty of the measurement in the value obtained.

Supplementary Note 5: Localised CO coordinates to normal modes

To compare the angle between the vibrational modes obtained from the T2DIR spectrum with that of the molecular components we need to translate the angle from localised CO coordinates of the calculated structure to normal modes. Supplementary Fig. 6 shows a schematic representation of the location of the normal modes of the CO groups of the macrocycle and $\mathrm{ni}^{\bullet-}$ station. The $\mathrm{ni}^{\bullet-}_{\mathrm{HB}}$ mode is the symmetric combination of the CO-stretch vibration of both CO groups. The transition dipole of the $\mathrm{ni}^{\bullet-}_{\mathrm{HB}}$ therefore lies on the bisector of the two C=O bonds (green arrow, Supplementary Fig. 6a). The transition-dipole moment of the amide I mode of an amide group arises predominantly from the carbonyl-stretch vibration and contains some contribution of the CN-stretch mode. The mixing causes the transition-dipole moment to deviate from the C=O bond by 20° in the direction C-N bond[10] (green arrow, Supplementary Fig. 6b). The angle between the $\mathrm{mc}_{\mathrm{HB}\cdots\mathrm{ni}^{\bullet-}}$ and $\mathrm{ni}^{\bullet-}_{\mathrm{HB}}$ modes obtained from the measurement is θ_{ij} in Supplementary Fig. 6c.

Supplementary Note 6: Cross sections of the T2DIR spectrum of the molecular shuttle at t_{UV} = 1,000 ns

Raw cross-sections. The uncorrected cross sections of the T2DIR spectrum of the molecular shuttle at t_{UV} = 1,000 ns are shown in Supplementary Fig. 8. Supplementary Fig. 8a shows the T1DIR spectrum of the molecular shuttle at t_{UV} = 1,000 ns. In addition to the bands discussed in the main text, we observe a positive change in absorption at ν_{probe} = 1,678 cm^{-1} which belongs to the $\mathrm{succ}_{\mathrm{free}}$ amide I band.

Depletion correction. In order to determine the overall scale factor of the ground-state spectrum that needs to be added to the excited-state spectrum, we use the diagonal peak of the ni_{s} mode when it is selectively pumped. The UV-induced bleach of the ni_{s} (at 1,700 cm^{-1}) is spectrally isolated and therefore purely reflects the depletion of the ground state[11]. In this cross section of the T2DIR and 2DIR spectra, the result of the addition should be zero across all frequencies because by selectively exciting the ni_{s} mode we should only observe the couplings associated with the ni station. We use the absorption change at 1,700 cm^{-1} to determine that 18.75 % of the ground state is depleted by the UV-pump pulse.

Supplementary Discussion

DFT calculations with different functionals and basis sets

In Supplementary Table 2 we compare the IR frequencies in the investigated spectral ranges as predicted by DFT calculations with different functionals and basis sets. In the model system (see Figure 4 of the main text), all features are included which we found to have an effect on the calculated frequencies of the vibrations of interest. We obtain good agreement between the experimental and calculated data (see next section). Furthermore, we find that the calculations with the B3LYP and B97D functionals using the 6-311G(d,p) basis set reproduce the experimental vibrational frequencies only slightly better than the B3LYP/6-31G(d) calculations. The results of the M062X/CC-pVDZ calculation are in substantially worse agreement with experiment. We can link the computed and experimental frequencies in order to achieve the best possible agreement after simple linear scaling (scaling factor 0.973).

Comparison of calculated and experimental transient spectra

In Supplementary Fig. 5a we compare the calculated transient 1D spectrum after charging the ni station with the experimental transient 1D spectrum (t_{UV} = 50 ns). In Supplementary Fig. 5b we compare the calculated transient 1D spectrum after shuttling (i.e., with the macrocycle hydrogen-bonded to the ni$^{\bullet-}$ station), and compare it with the experimental transient 1D spectrum at t_{UV} = 4000 ns. The calculated and experimentally observed IR frequencies are listed in Supplementary Table 2. The good agreement supports the boat-like co-conformation in the final state, which was also derived from the T2DIR spectrum.

Supplementary References

1. Chung, H. S., Khalil, M., Smith, A. W. & Tokmakoff, A. Transient two-dimensional IR spectrometer for probing nanosecond temperature–jump kinetics. *Rev. Sci. Instr.* **78,** 063101-01–063101-10 (2007).
2. Krejtschi, C. & Hauser, K. Stability and folding dynamics of polyglutamic acid. *Eur. Biophys. J.* **40,** 673–685 (2011).
3. Huerta-Viga, A., Shaw, D. J. & Woutersen, S. pH dependence of the conformation of small peptides investigated with two-dimensional vibrational spectroscopy. *J. Phys. Chem. B* **114,** 15212–15220 (2010).
4. Haldrup, K. Singular value decomposition as a tool for background corrections in time-resolved XFEL scattering data. *Phil. Trans. R. Soc. B* **369,** 20130336 (2014).
5. Rousseeuw, P. J. & Croux, C. Alternatives to the Median Absolute Deviation. *J. Am. Stat. Assoc.* **88,** 1273–1283 (1993).
6. Golonzka, O., Khalil, M., Demirdöven, N. & Tokmakoff, A. Vibrational Anharmonicities Revealed by Coherent Two-Dimensional Infrared Spectroscopy. *Phys. Rev. Lett.* **86,** 2154–2157 (10 2001).
7. Lavalette, D., Tétreau, C., Tourbez, M. & Blouquit, Y. Microscopic viscosity and rotational diffusion of proteins in a macromolecular environment. *Biophys. J.* **76,** 2744–2751 (1999).

8. Horng, M., Gardecki, J. & Maroncelli, M. Rotational dynamics of coumarin 153: time-dependent friction, dielectric friction, and other nonhydrodynamic effects. *J. Phys. Chem. A* **101,** 1030–1047 (1997).

9. Brouwer, A. M. *et al.* Photoinduction of fast, reversible translational motion in a hydrogen-bonded molecular shuttles. *Science* **291,** 2124–2128 (2001).

10. Hamm, P. & Zanni, M. T. *Concepts and Methods of 2D Infrared Spectroscopy* (Cambridge University Press, 2011).

11. Panman, M. R. *et al.* Operation mechanism of a molecular machine revealed using time-resolved vibrational spectroscopy. *Science* **328,** 1255–1258 (2010).
